# Signalling through RXRβ and its agonist, bexarotene, promotes neuron formation in *Xenopus laevis* embryos

**DOI:** 10.1242/bio.062675

**Published:** 2026-07-16

**Authors:** Daniela Lopes Cardoso, Marilena Loizou, Danai D. Fourla, Charles Woodman, Matthew J. Guille, Colin Sharpe

**Affiliations:** ^1^ School of Environment and Life Sciences, Institute of Life Sciences and Healthcare, University of Portsmouth, Portsmouth PO1 2DT, UK; ^2^ European Xenopus Resource Centre, School of Environmental and Life Sciences, University of Portsmouth, Portsmouth PO1 2DT, UK

**Keywords:** *Xenopus*, Retinoid, Bexarotene, Neurogenesis, Embryonic primary neuron

## Abstract

Retinoic acid, acting through RAR/RXR nuclear receptors, is required for neuronal differentiation in *Xenopus laevis*. Bexarotene, characterised as a pan-RXR agonist, reduces the symptoms of Alzheimer's disease in mouse models by clearing amyloid plaque and by promoting neurogenesis. In this paper, we show that RXR-initiated bexarotene signalling generates additional neurons both during normal *X. laevis* development and in ectodermal explants in which bone morphogenetic protein (BMP) signalling is inhibited. Differential gene expression analysis between ectodermal explants taken from uninjected embryos and from embryos co-injected with mRNA that expresses Noggin, a BMP antagonist, and RXRβ identifies genes mediating the transition from an epidermal to a neural fate. The explants express genes associated with an anterior neural fate, but not a neuronal fate. The addition of bexarotene to equivalent co-injected explants activates genes that promote neuronal differentiation and posterior character, including genes of the canonical Wnt signalling pathway. *Xenopus* ectodermal explants therefore provide a simple and efficient tool to identify novel retinoid agonists that promote neuronal differentiation. The genes expressed in response to bexarotene are consistent with a pathway to neuronal differentiation allied to standard retinoid signalling.

## Teaser

**Summary:** Bexarotene, an RXR agonist that ameliorates Alzheimer's disease symptoms in mouse models, drives neuronal differentiation in *Xenopus* embryos and explants by activating a pathway closely related to standard retinoid signalling.

## INTRODUCTION

Bexarotene is an agonist for the RXR nuclear receptor that, in mammals, can activate genes associated with neurogenesis (Boehm et al., 1995; Lefterov et al., 2015; Mounier et al., 2015; Nam et al., 2016) and, when added to mammalian adult neural stem cells in culture, increases their proliferation and subsequently promotes late-stage neuronal differentiation (Saibro-Girardi et al., 2024). Bexarotene has been proposed to ameliorate the symptoms of Alzheimer's disease in mouse models (Cramer et al., 2012), possibly through the clearance of pathogenic levels of plaque, though this remains uncertain (Landreth et al., 2013; Tousi, 2015). An understanding of the function of RXR-bexarotene signalling, particularly in neuron formation, may have relevance in the context of neurodegenerative disorders.

Like most vertebrates, the genome of *Xenopus tropicalis* encodes three retinoic acid receptors (RARα, β, and γ) and three retinoid X receptors (RXRα, β, and γ), though these genes are duplicated in the allotetraploid *Xenopus laevis* (Blumberg et al., 1992). The RAR nuclear receptors predominantly function as heterodimers with an RXR protein to influence the expression of target genes (Evans and Mangelsdorf, 2014). Retinoid signalling in *Xenopus* embryos affects processes including anterior-posterior (AP) patterning (Durston et al., 1989; Papalopulu and Kintner, 1996) and neurogenesis (Sharpe and Goldstone, 2000a,b, 1997). In mammals, retinoid signalling, through the addition of *all-trans* retinoic acid (*at*RA), is a key step in the conversion of cultured embryonic stem cells to a neuronal fate (Kim et al., 2009; Schuldiner et al., 2001; Shi et al., 2012a,b; Strickland and Mahdavi, 1978).

*X. laevis* rapidly develops a set of embryonic primary neurons (EPNs) that become active within a few days following fertilisation (Hartenstein, 1993; Lamborghini, 1980) to coordinate basic swimming movements (Roberts et al., 2010). The EPNs are similar in form and function to later-developing neurons and, given their rapid development, small numbers and dependence on common developmental pathways, provide an excellent tool to examine vertebrate neurogenesis.

Retinoid signalling promotes the formation of EPNs in *X. laevis* (Sharpe and Goldstone, 2000a). The critical time for retinoid signalling is across the early gastrula stages, and inhibition of retinoid signalling results in smaller expression domains for the proneural gene neurogenin-2 (*neurog-2*) and fewer EPNs (Sharpe and Goldstone, 2000a). Isolated animal cap (pre-gastrula ectodermal sheet) explants, in which bone morphogenetic protein (BMP) signalling has been inhibited by ectopic Noggin expression form neural epithelia but do not, however, form differentiated neurons. This ability can be conferred by retinoid signalling (Sharpe and Goldstone, 2000b).

Here, we demonstrate that enhanced RXRβ-bexarotene signalling in *X. laevis* embryos results in an increase in the number of observed neurons. The ability to promote neuron formation in Noggin-treated animal cap explants is used to identify genes whose expression is enhanced in response to RXRβ-bexarotene signalling in this system.

This approach confirms that Noggin application restricts the formation of a mucociliary epidermis and activates the expression of transcription factors that promote the formation of neural tissue of an anterior character, but without neuronal differentiation. The concurrent activation of RXRβ-bexarotene signalling represses the expression of these transcription factors, activates genes of the Wnt signalling pathway and promotes the expression of transcription factors required for the differentiation of neural precursor cells to neurons. The activation of the *rara* gene in this process suggests a mechanism for the promotion of neuron formation by endogenous retinoids and bexarotene.

## RESULTS

### *X. laevis* animal cap explants identify a panel of indicator genes activated by retinoid signalling

**Fig. 1.**
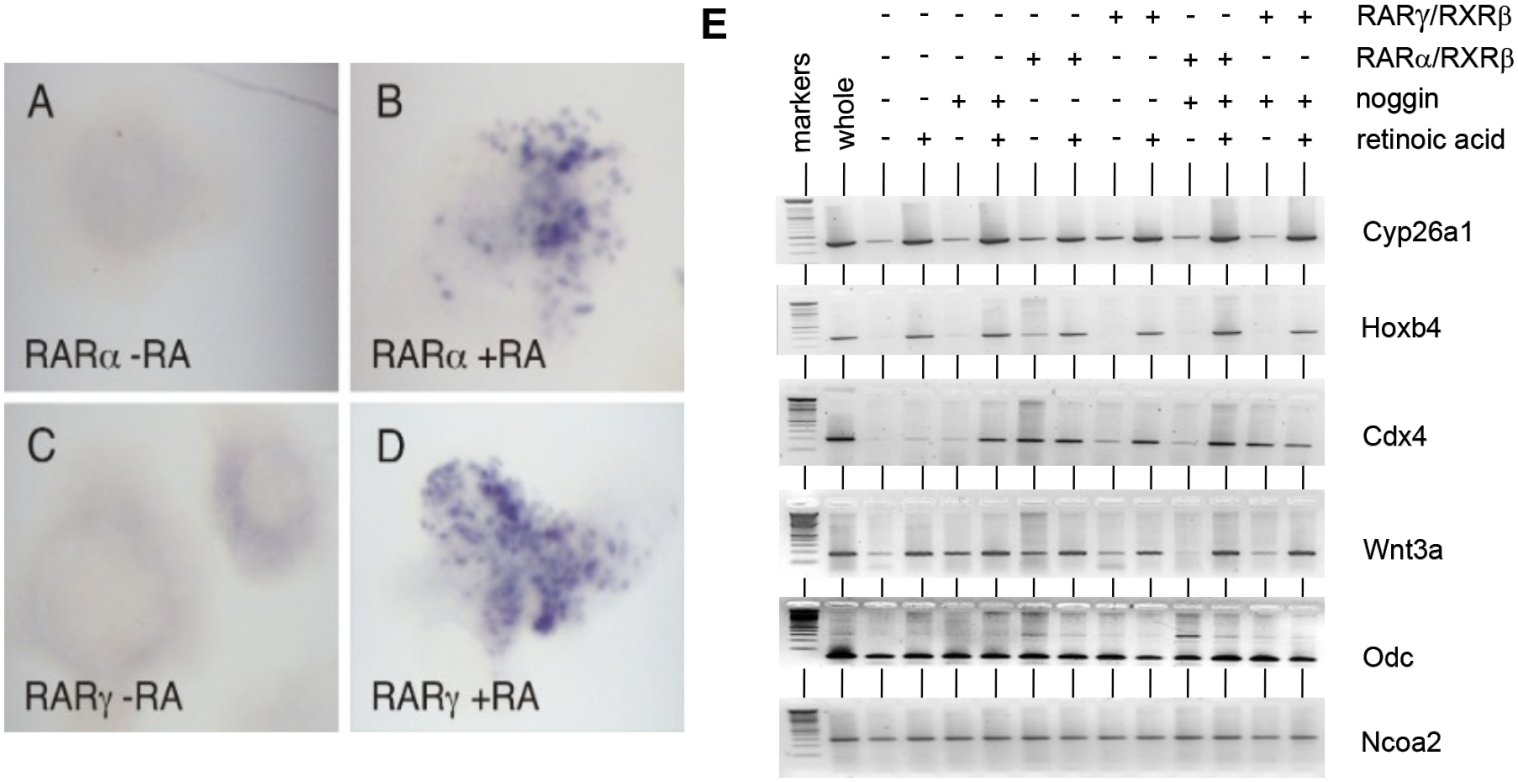
**Heterodimers of either RARα or RARγ with RXRβ generate neurons in Noggin-treated animal caps.** (A-D) Animal cap pairs removed from embryos injected with Noggin and RXRβ mRNAs and either RARα (A,B) or RARγ (C,D) mRNA and grown either in 1× MBS (A,C) or 1× MBS+100 nM *at*RA to the equivalent of the late neurula stage. *In situ* hybridisation with an antisense RNA probe against a neural-specific type III beta tubulin identifies neurons in the samples grown with *at*RA (B,D). (E) Representative example of an RT-PCR analysis of RNA from embryos and animal cap explants injected with synthetic RARα, RARγ, RXRβ and Noggin mRNAs as described above the image and grown with and without 100 nM *at*RA to the late neurula stage. The expression of specific genes is labelled. *Odc* and *Ncoa2* represent genes whose level of expression is unaffected by retinoid signalling.

In *Xenopus*, EPNs are derived from ectodermal cells, though animal cap explants isolated at the late blastula stage [Nieuwkoop and Faber (NF) stage 9] do not develop into differentiated EPNs. A basic requirement for neuron formation is the inhibition of BMP signalling within the animal cap, achieved experimentally by injecting Noggin mRNA into the early embryo (Sharpe and Goldstone, 2000a). Retinoid signalling through RARα/RXRβ is then sufficient to promote neuronal differentiation in Noggin-expressing explants (Sharpe and Goldstone, 2000a,b). The RARγ isotype is also expressed in the early embryo, and when Noggin-mRNA-injected animal caps are co-injected with RARγ/RXRβ mRNA and grown in *at*RA from the early gastrula stage, they also undergo neurogenesis (Fig. 1A-D). Reverse transcriptase PCR (RT-PCR) using these explants identifies the activation of two RA-responsive genes upregulated through defined retinoic acid response elements (RAREs), *hoxb4* (Gould et al., 1998) and *cyp26a1* (Zhang et al., 2010), and two genes known to interact with retinoid signalling, *cdx4* (Houle et al., 2000) and *wnt3a* (Shum et al., 1999) (Fig. 1E). Expression of these four genes provides a basic indicator of signalling through the characterised RAR/RXR-*at*RA signalling pathway and indicates a requirement for retinoid signalling that is not dependent on the RAR isotype.

### Each of the two major RXRβ protein isoforms expressed during early *X. laevis* development mediates bexarotene-dependent neuronal differentiation

**Fig. 2.**
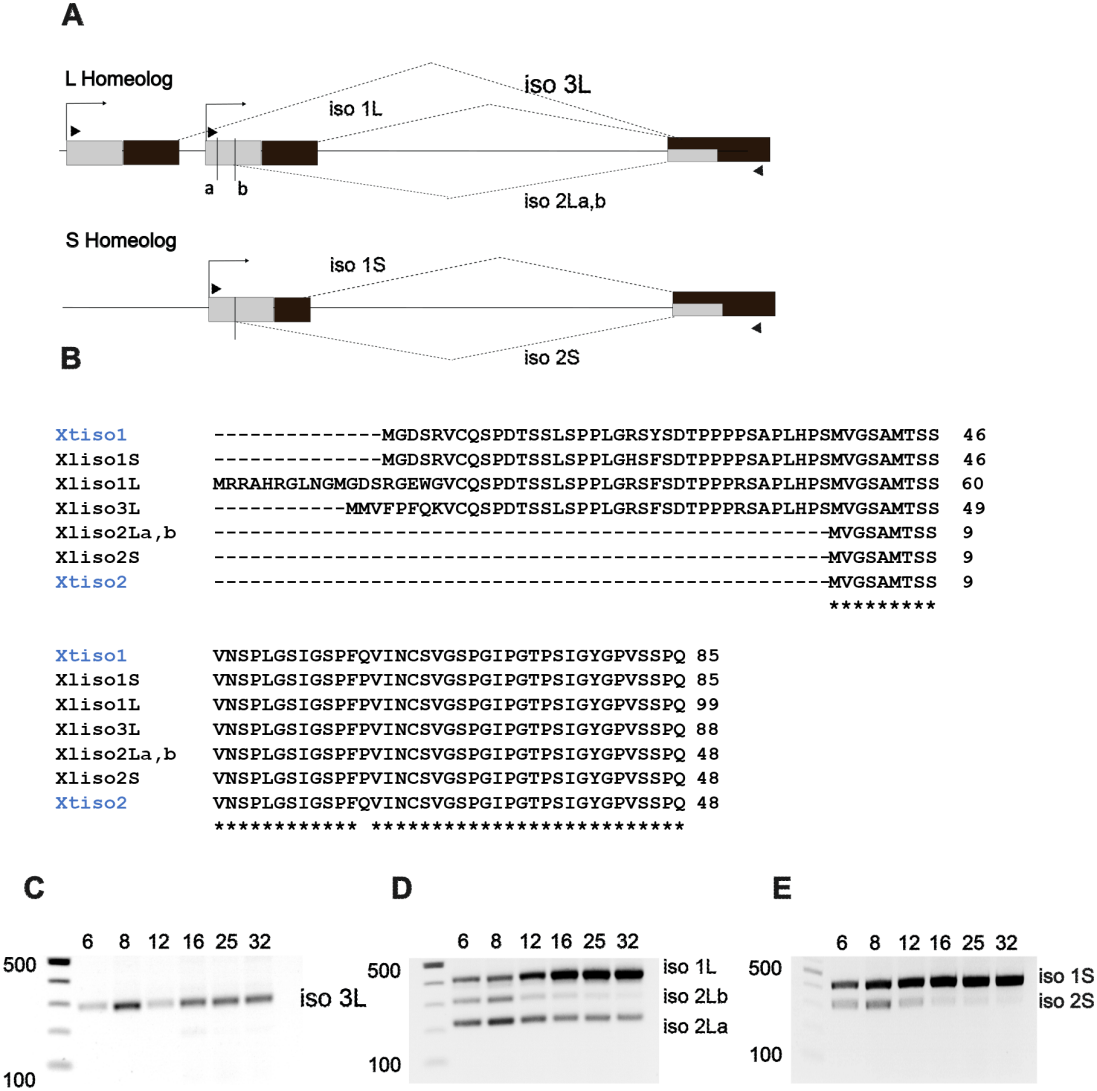
***X. laevis* RXRβ generates multiple transcript isoforms.** (A) Mapping the 5′ end of the RXRβ *X. laevis* homeologs predicts that the two genes generate at least six major transcript isoforms that differ at the 5′ end. Horizontal arrows are transcriptional start sites, and boxes mark exons. Grey shading indicates a 5′ untranslated region, whilst black boxes contribute to the protein sequence. The beginning of L homeolog exon 3 and S homeolog exon 2 contributes an untranslated sequence to isoform 2 transcripts and a protein-coding sequence to isoform 1 and 3 transcripts. Vertical lines a and b in exon 2 of homeolog L represent internal splice donor sites, and the arrowheads indicate the PCR primers used in sections C-E. (B) Alignment of RXRβ protein isoform N-terminal sequences from *X. laevis* and *Xenopus tropicalis*. Two protein isoforms are conserved between diploid *X. tropicalis* and pseudo-tetraploid *X. laevis*. (C-E) Expression of the RXRβ transcripts across early development. Representative RT-PCR using primers from specific regions of each transcript (arrowheads in Fig. 2A) against cDNA generated from embryos across early developmental stages (NF developmental stages, where stage 12 is mid-gastrula) shows that all transcripts have a maternal component and expression is maintained at least until the swimming tadpole (stage 32), though the levels of iso2Lb and iso2S decline after the gastrulation stages, repeated from two independent batches of embryos.

Since the RXR agonist, bexarotene, promotes aspects of neuronal differentiation in mammalian models (Mounier et al., 2015; Saibro-Girardi et al., 2024), the next aim is to assess whether RXRβ-bexarotene signalling, like the related RAR/RXR-*at*RA signalling, can similarly promote neuronal differentiation during *Xenopus* development. *X. tropicalis* RXRβ (Xenbase: XB-GENE-480167; https://www.xenbase.org/xenbase/, NCBI Gene ID: 548691; https://www.ncbi.nlm.nih.gov/gene/) has two predominant isoforms: NP_001015937 and a second isoform that is 37 residues shorter at the amino-terminus (e.g. XP_012823808). These isoforms are present in *X. laevis* (Fig. 2A) along with two additional protein isoforms from the L homeolog of the pseudotetraploid *X. laevis* genome (Xliso 1L and 3L) (Fig. 2B-E). Given the conservation of the long and short protein isoforms between *X. laevis* and *X. tropicalis*, the activity of these (iso1S and iso2S) is analysed further. Since the amino-terminal domains of nuclear receptors are associated with transcriptional activation (Folkers et al., 1995), can both isoforms drive neuronal differentiation?

**Fig. 3.**
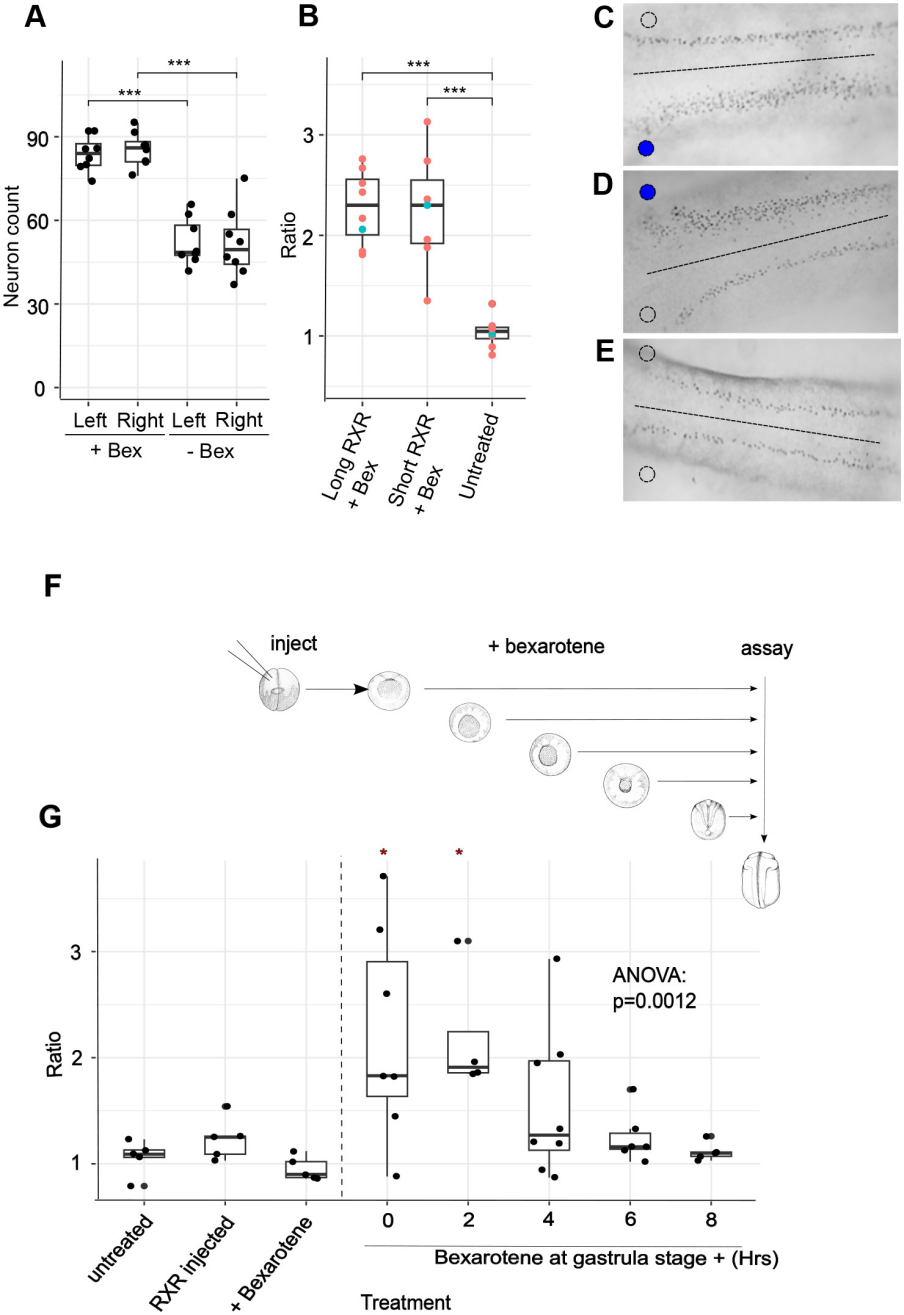
**Signalling through RXRβ isoforms, at the early gastrula stage, promotes the formation of primary embryonic neurons.** (A) Embryos treated with 100 nM bexarotene have more Isl-1-positive EPNs than untreated embryos at the early tailbud stage (*n*=8 embryos per treatment). A *t*-test identifies a significant increase in neurons between treatments where *** is *P*<0.001. (B-E) Quantitative analysis of Isl-1-positive EPNs indicates that embryos injected with either RXRβ iso1 or iso2 and treated with bexarotene generate a more than twofold increase in these neurons on the injected side (*n*=7 embryos per treatment, analysis of variance with Tukey's honestly significant difference test). Points in blue on the dot plot correspond to examples C-E. (C) Dorsal view of iso2-injected embryo, (D) iso1-injected embryo, (E) untreated embryo. Dashes mark the dorsal midline. (F) Embryos are injected into 1 cell at the two-cell stage with RXRβ and β-galactosidase mRNA. Bexarotene is added to a batch of the injected embryos at stage 10 and then to separate batches at 2-h intervals at 21°C, corresponding approximately to stages 10.5, 11, 12.5 and 14. All embryos are then grown to stage 22 before being fixed. (G) In the first three samples, embryos are either untreated, injected with RXR and β-galactosidase mRNA at the two-cell stage or uninjected embryos are given bexarotene at stage 10. Embryos are then processed for immunohistochemistry with the anti-Isl1 monoclonal antibody (*n*>5 embryos for each treatment or timepoint). Stained nuclei are counted on the injected (βgal positive) and uninjected sides, and the ratios are plotted. An analysis-of-variance statistical test indicates differences between the samples, and Tukey's honestly significant difference post hoc test identifies a significant increase in neurons on the injected side (where * is *P*<0.05) following the addition of bexarotene at stages 10 and 10.5.

Incubating embryos at the early gastrula stage in 100 nM bexarotene results in the formation of additional Isl-1-positive EPN sensory neurons compared to untreated embryos (Fig. 3A). Injecting synthetic *X. laevis* mRNA encoding either the short or long protein isoform of RXRβ into one cell of the two-cell embryo results in an increase in Isl-1-positive cells on the injected compared with the uninjected side, when embryos are grown in bexarotene from the early gastrula stage (Fig. 3B-E). These results demonstrate that, in *X. laevis*, bexarotene-RXR signalling, through either the short or long isoforms of RXRβ, promotes the formation of additional EPNs.

To identify the latest developmental stage when enhanced RXR-bexarotene signalling is required to generate additional neurons, embryos are injected with RXRβ mRNA (iso2S) at the two-cell stage, and bexarotene is subsequently added to groups of embryos at intervals across the gastrula stages (Fig. 3F). When assayed at the early tailbud stage, only those embryos that had received bexarotene at the early gastrula stage show an asymmetric distribution of Isl-1-positive cells, with more neurons on the injected side of the embryo (Fig. 3G). Embryos receiving bexarotene later than stage 11 make only marginally more EPNs (Fig. 3G). This demonstrates that, as seen previously for RAR/RXR-*at*RA signalling (Sharpe and Goldstone, 2000a), enhanced RXRβ-bexarotene signalling is required in the early gastrula embryo.

### RXRβ signalling promotes neuron formation in noggin-neuralised, isolated animal caps

**Fig. 4.**
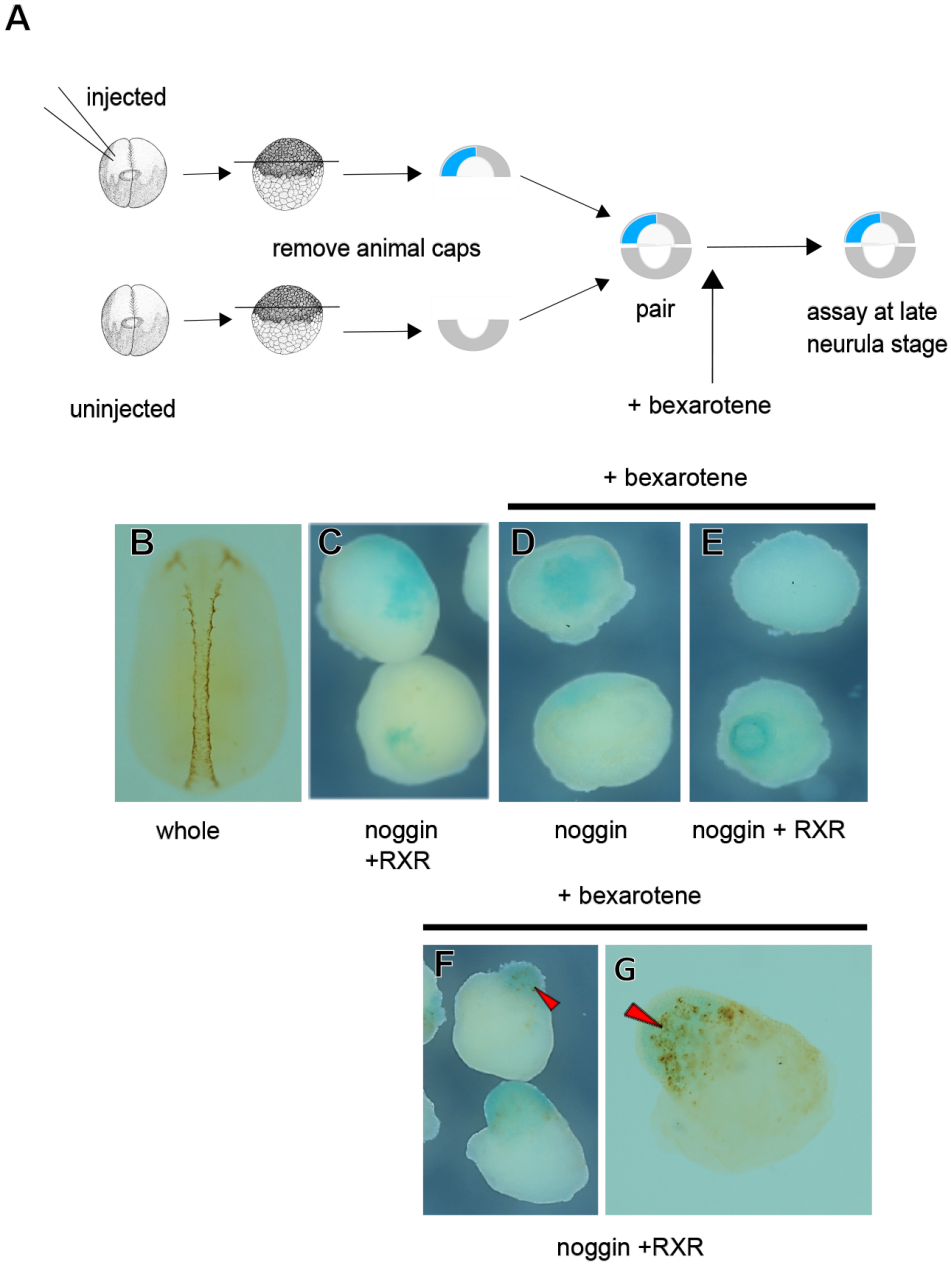
**Enhanced RXRβ signalling generates EPNs in noggin-neuralised animal caps.** (A) Embryos are injected into one cell with combinations of Noggin, RXRβ and β-galactosidase mRNA at the two-cell stage. At NF stages 9 and 10, animal caps are removed from injected and uninjected embryos, formed into pairs and grown in the presence, or absence, of 100 nM bexarotene to the equivalent of the late neurula stage and fixed for antibody staining, *n*=4 animal cap pairs per treatment. (B) Late-neurula-stage embryo (anterior at the top of the picture) HRPO stained with the anti-HNK-1 antibody. (C-G) Representative animal cap explants stained for β-galactosidase expression (blue) and NCAM with the anti-HNK-1 antibody (brown). Staining consistent with neurons is seen only in animal caps injected with both noggin and RXRβ and then treated with bexarotene (compare C with F and G). Animal cap explant in G has been rendered transparent in Murray's clear to make the stained cells (red arrowhead) more apparent. HNK-1 staining is found only in the region derived from the injected animal cap.

To determine whether RXR-bexarotene signalling has the capacity to promote EPN formation in animal caps, embryos are injected into one cell at the two-cell stage with synthetic RXRβ and Noggin mRNA and grown to the late blastula stage. Animal caps taken from injected embryos are then paired with uninjected caps and grown as explants, with or without bexarotene. When embryos, grown under the same conditions, reached stage 22, the explants are fixed and assayed using an anti-HNK-1 monoclonal antibody, which in *X. laevis* primarily recognises an neural cell adhesion molecule (NCAM) epitope on axons and in the Golgi apparatus of neuronal cell bodies (Nordlander, 1993) and in some neural crest derivatives (Fig. 4A). Only animal caps injected with both Noggin and RXRβ mRNA and grown in bexarotene show HNK-1 staining consistent with neuronal differentiation (Fig. 4B-G). Consequently, RXR-bexarotene signalling has the same ability as RAR/RXR-*at*RA signalling to promote EPN formation in noggin-treated animal caps and is required across similar developmental stages.

### Transcriptome analysis of isolated ectodermal explants identifies four responses to the expression of Noggin and RXRβ mRNAs in explants

In this section, RNA-seq analysis is used to catalogue genes differentially expressed in response to bexarotene-RXR signalling. Using animal cap explants restricts the responses to events in ectodermal cells, providing a focus on the enhanced formation of EPNs.

**Fig. 5.**
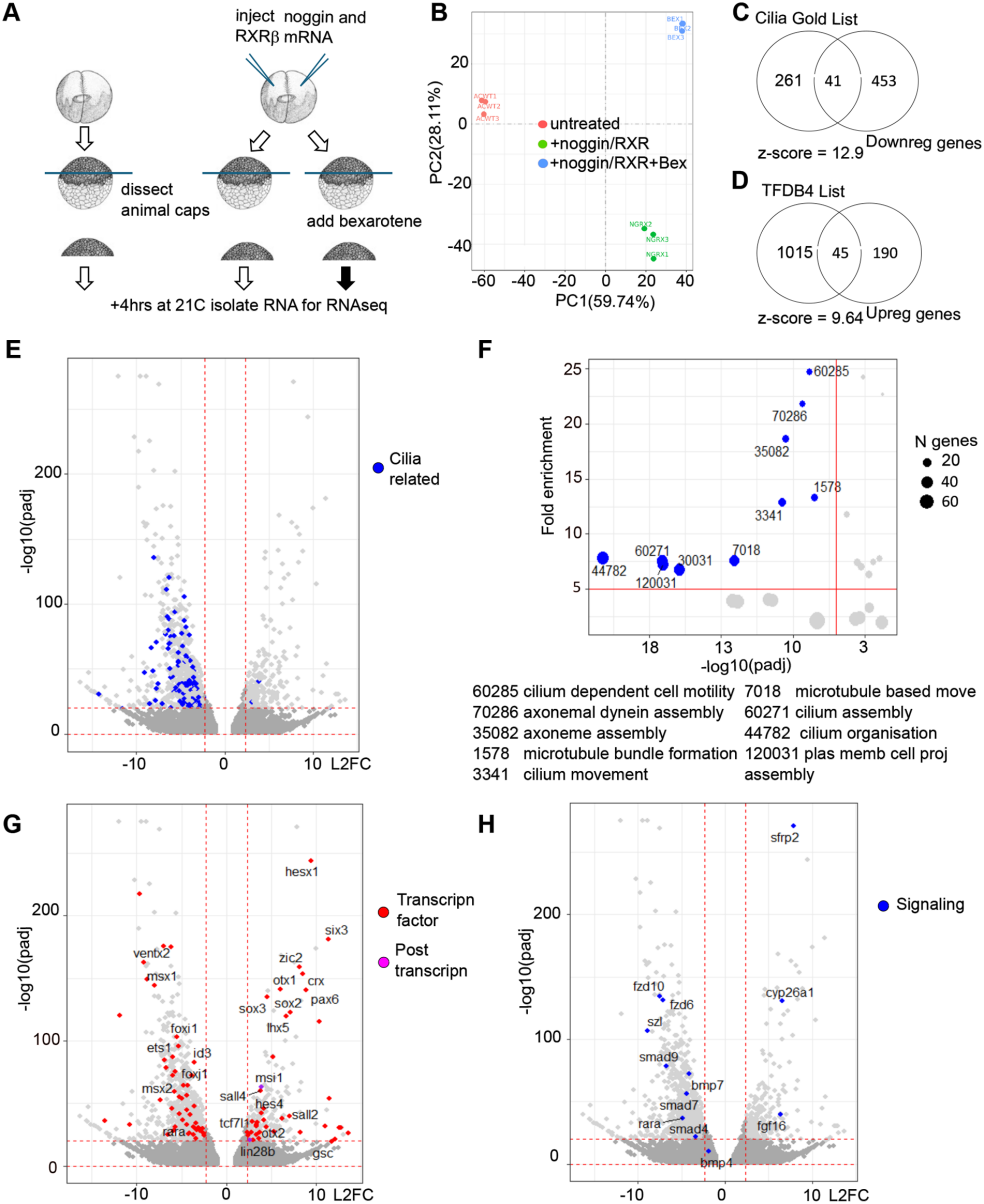
**Animal cap explants from embryos injected with Noggin and RXRβ mRNA downregulate genes associated with a mucociliary epidermal fate and upregulate genes associated with an anterior neural fate.** (A) Outline of the protocol for the preparation of the reprogrammed animal cap explants. (B) Principal component analysis shows distinct characteristics for each of the three treatments. Three pairs of animal caps were used to generate one sample, and this was repeated two more times to generate three replicates for each of the treatments. (C) A comparison of the downregulated genes and the CiliaCarta Gold list of genes shows a greater-than-expected overlap, confirming the action of noggin in antagonising a mucociliary epidermal fate. (D) A comparison of upregulated genes and a database of genes that affect transcription (AnimalTFDB4) indicates a greater-than-expected overlap, confirming that the activation of transcription factors plays a role in the cell fate changes in the explants. (E) Volcano plot of upregulated and downregulated genes in animal cap explants in response to the injection of noggin and RXRβ mRNA. Genes found in the CiliaCarta Gold list are highlighted in blue. The vast majority are found in the downregulated section of the plot. (F) GO-term over-representation dot plot for all significant downregulated genes predominantly identifies terms associated with cilia and associated structures. (G) Same volcano plot as in E, but with genes associated with transcriptional control of gene expression highlighted in red. Key genes mentioned in the text are labelled. Two genes affecting post-transcriptional aspects of gene expression are shown in mauve. (H) Same volcano plot as in E but with genes associated with intercellular signals highlighted in blue. Genes mentioned in the text are labelled.

**Fig. 6.**
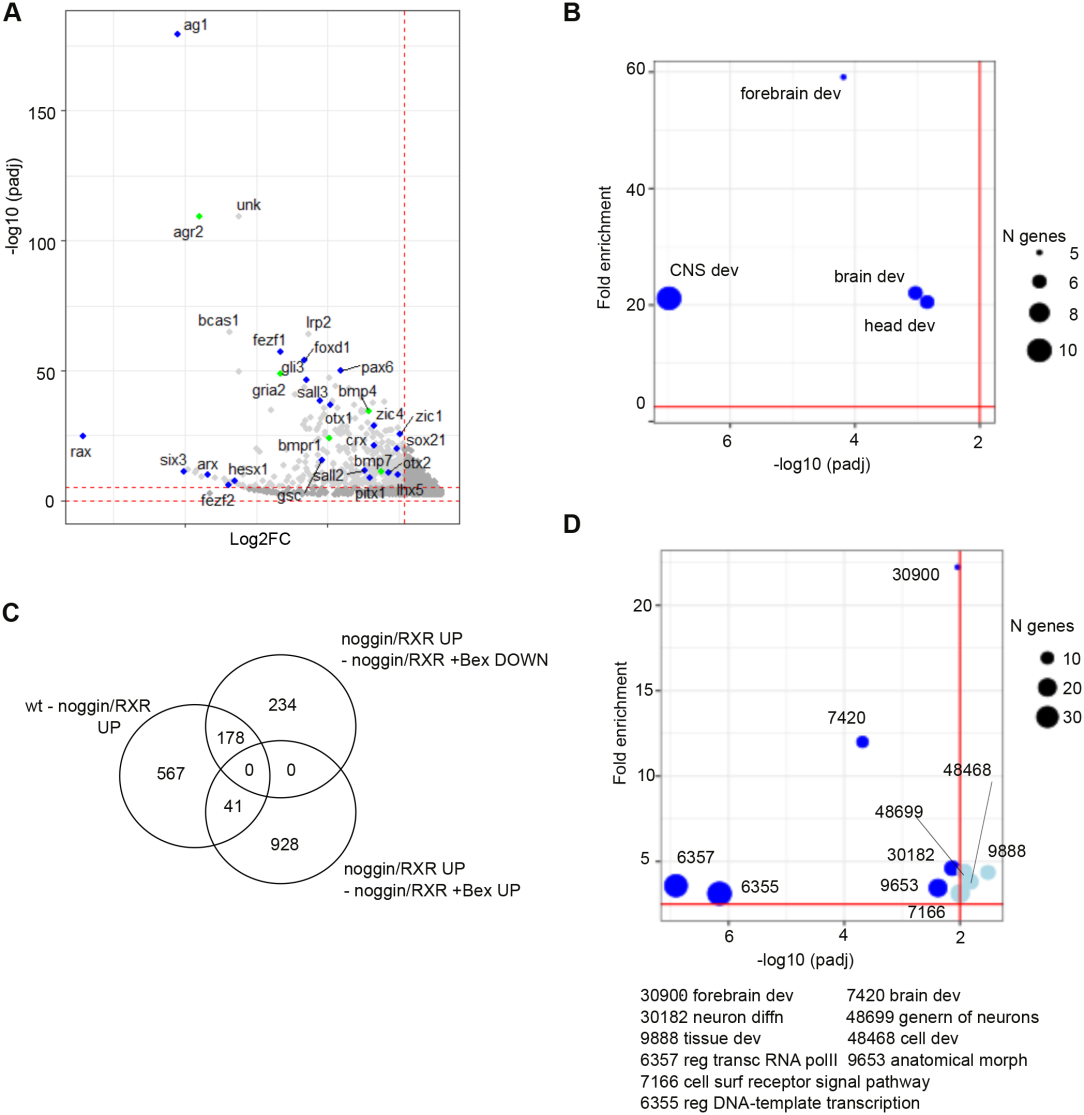
**Analysis of genes downregulated in noggin-neuralised explants in response to enhanced RXR-bexarotene signalling.** (A) Volcano plot of downregulated genes. Light grey dots represent genes that meet the significance criteria, and dark grey dots represent those below the described criteria. Blue dots are transcription factor genes mentioned in the text, and green dots are genes involved in intercellular signalling. (B) Biological process GO-term dot plot of the downregulated transcription factors that meet the significance criteria identifies genes involved in anterior neural development. (C) Venn diagram of three sets of genes. The left-hand circle represents genes upregulated at least fivefold in Noggin and RXR mRNA-injected explants in the absence of bexarotene. The upper-right circle is genes downregulated at least fivefold in Noggin and RXR-mRNA-injected explants in the presence of bexarotene. There are 178 genes that were upregulated in the absence of bexarotene but downregulated in its presence. The lower-right circle represents genes upregulated in Noggin and RXR-mRNA-injected explants in the presence of bexarotene, and there are 41 genes upregulated in both the absence and further in the presence of bexarotene. There are 567 genes upregulated in response to Noggin and RXR mRNA injection in the absence of bexarotene that are predominantly unaffected by the addition of bexarotene. (D) Biological process GO-term dot-plot analysis of the 178 genes upregulated in the absence of bexarotene but downregulated in its presence relates predominantly to neural development and RNA polII transcription.

**Fig. 7.**
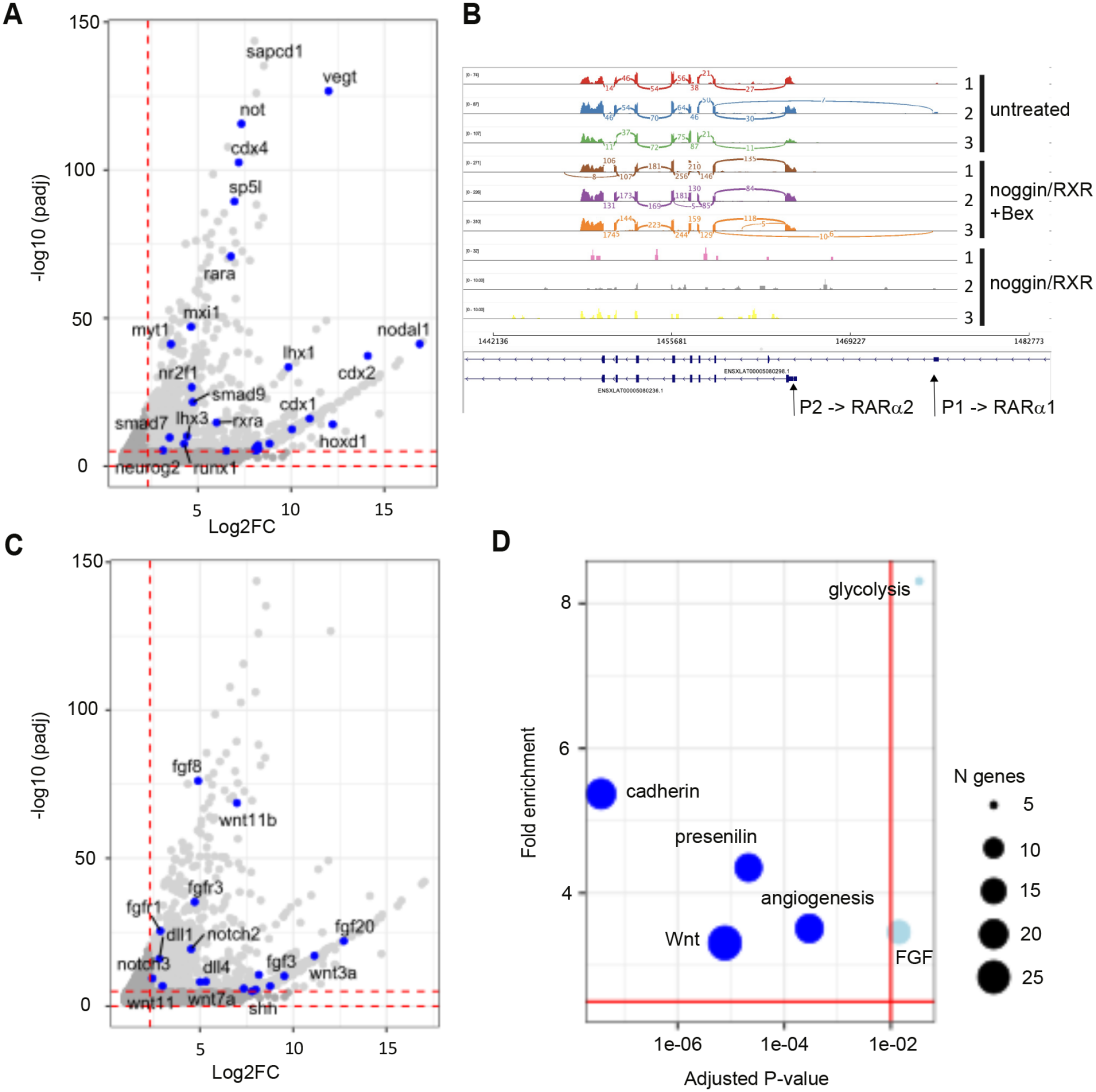
**Analysis of genes upregulated in Noggin-neuralised explants in response to enhanced RXR-bexarotene signalling.** (A) Volcano plot of upregulated genes in noggin-neuralised explants in response to enhanced RXR-bexarotene signalling where those meeting the imposed significance threshold are in light grey and those outside these limits are in dark grey. Genes related to transcription are highlighted in blue, and those mentioned in the text are labelled. (B) Sashimi plot of transcript density at the RARα gene locus. Compared to untreated animal caps, the injection of Noggin and RXRβ mRNA decreases overall expression of the RARα gene. The addition of bexarotene to the Noggin and RXRβ-mRNA-injected animal caps results in an increased expression of the RARα gene from the P2 promoter, generating the RARα2 isoform. The P2 promoter contains a DR-5 RARE. (C) The same volcano plot as in A but with genes related to intercellular signalling mentioned in the text highlighted. (D) Dot plot of the PANTHER Pathways analysis identifies Wnt and FGF signalling pathways. Many of the genes in the cadherin and presenilin sets overlap with the genes in the Wnt signalling set.

The transcriptomes derived from untreated animal caps are first compared to those derived from Noggin and RXRβ-mRNA-co-injected embryos (Fig. 5A). In the absence of bexarotene, RXRβ may have a limited repressive effect on gene expression, but this treatment does not result in the differentiation of EPNs (Fig. 4C). Consequently, RXRβ mRNA was co-injected along with Noggin mRNA so that, in a second analysis, the transcriptomes from Noggin- and RXRβ-mRNA-co-injected animal caps grown without and with bexarotene can be compared (Figs 6,7). In all cases, the explants are maintained until embryos from the same batch reach mid-gastrula (NF stage 12) (Fig. 5A). The final group of animal caps therefore receive enhanced RXRβ-bexarotene signalling across the critical period determined above (Fig. 4) and are investigated at the end of this period, but before the appearance of differentiated neurons. Biological replicates clustered tightly by condition (Fig. 5B), indicating high reproducibility (details of the RNA-seq analysis are presented in Table S1).

In contrast to untreated animal cap explants, which form a mucociliary epithelium (Angerilli et al., 2018), the presence of Noggin inhibits BMP signalling, resulting in the formation of neural tissue (Chitnis and Kintner, 1995). These explants, nevertheless, lack the ability to differentiate into neurons (Fig. 4C) (Sharpe and Goldstone, 2000b). Comparing the selected, differentially expressed genes (DEGs) (Table S2) with the CiliaCarta Gold list of genes (Van Dam et al., 2019) involved in cilia formation and function shows an overrepresentation of these genes in the downregulated set (Fig. 5C,E). Biological process GO-term overrepresentation analysis of the downregulated DEGs also predominantly identifies terms linked to cilia formation and function (Fig. 5F).

By examining the DEGs after just 4 h, we aim to identify the genes that drive a shift towards a neural fate. A comparison of the DEG list to the AnimalTFDB4 list of genes that mediate gene expression (Shen et al., 2023) again shows an overrepresentation (Fig. 5D,G). Amongst those downregulated are direct targets of BMP signalling, including *Foxj1*, a transcription factor that directs the formation of a ciliated epithelium (Vij et al., 2012), and *Foxi1*, which directs the formation of ionocytes (Bowden et al., 2026). Together, these factors drive the development of the *Xenopus* mucociliary epithelium. *Ventx2*, a direct target of BMP signalling that represses neural development (Schuler-Metz et al., 2000; Taylor et al., 2006), is also downregulated, along with *Msx1/2* (Khadka et al., 2006; Suzuki et al., 1997; Tríbulo et al., 2003), *Ets1* and its direct target *Id3*, which promotes neural crest development and formation of the neural-epithelial border (Wang et al., 2015).

GO-term enrichment analysis of all upregulated DEGs (Fig. S1) identifies terms related to nervous system development, with emphasis on anterior structures. These include upregulated transcription factors that are predominantly involved in promoting an anterior neural state and include *hesx1* (Ermakova et al., 2007; Martynova et al., 2004), *sox2/3* (Rogers et al., 2008, 2009), *lhx5* (Toyama et al., 1995), *otx1/2* (Andreazzoli et al., 1997), *pax6* (Moreno et al., 2008; Nakayama et al., 2015) and *zic1-4* (Merzdorf and Forecki, 2018) (Fig. 5G; Table S3). Related to this activity, genes including *six3* (Gestri et al., 2005), *sox21* (Whittington et al., 2015) and *hes4* (El Yakoubi et al., 2012) promote the proliferation, rather than the differentiation, of neural precursor cells. In addition, *gsc* (Kumar et al., 2023) and *zeb2* (Nitta et al., 2004) contribute to the inhibition of BMP expression, whilst transcription factors, including *sall2* (Onai et al., 2004) and *tcf7l1* (Kuwahara et al., 2014), limit the activity of the Wnt signalling pathway that would otherwise contribute to posterior identity and neuronal differentiation.

Also upregulated in response to the inhibition of BMP signalling are Msi1, a protein that mediates translational control of gene expression implicated in cell fate determination (MacNicol et al., 2015), and lin28b, an RNA-binding protein that limits the activity of a specific subset of miRNAs involved in neural development (Corallo et al., 2020) and may act independently of this function in cell fate determination (Faas et al., 2013), suggesting that post-transcriptional mechanisms also contribute to the change from a mucociliary epithelial to a neural fate.

There are also changes in the expression of genes whose proteins encode intercellular signals (Fig. 5H). The levels of expression of *bmp4* and *bmp7*, the predominant BMP signals in the gastrula embryo, are downregulated along with *smad4*, though *smad7* and *smad9*, which are generally considered to inhibit BMP signalling, are also downregulated. A significant upregulation is also seen for *cyp26a1*, which encodes an enzyme that catabolises retinoic acid, though it may also generate other active retinoids (Sonneveld et al., 1999). Consistent with an inhibition of retinoid signalling, the expression of the retinoid receptor gene, *rara*, is also downregulated.

Significant changes are seen in components of the Wnt signalling pathway (Fig. 5H); notably, the expression of *sfrp2* (Kong et al., 2012), encoding an antagonist of canonical Wnt signalling, is upregulated, whilst Wnt receptor genes *fzd10* and *fzd6* are downregulated. In contrast, expression of the gene *szl*, which produces a secreted Wnt inhibitor, is downregulated, consistent with being a direct target of BMP signalling, though Szl may be more active in the non-canonical Wnt pathways (Yoon et al., 2024). The only notable change in fibroblast growth factor (FGF) signalling is seen with the increased expression of the *fgf16* gene, which has been implicated in the patterning of brain regions (Elsy et al., 2019).

In summary, explants from Noggin- and RXRβ-mRNA-co-injected embryos show at least four responses. First, BMP signalling is suppressed by promoting the expression of *gsc* and *zeb2*. Second, mucociliary epidermal development is prevented by downregulating the expression of transcription factors, notably *foxi* and *foxj*. Third, neural development is promoted by downregulating the expression of *ventx2*. And fourth, a proliferative neural precursor state is promoted through enhanced expression of transcription factor genes such as *six3*, *sox21* and *hes4*, with a predominantly anterior character through the repression of the posteriorising Wnt signalling and upregulation of genes including *hesx1*, *sox2*, *pax6*, *otx1* and *otx2*.

### Noggin-neuralised animal caps with enhanced RXRβ-bexarotene signalling downregulate genes associated with anterior neural development

Explants grown from Noggin- and RXRβ-mRNA-injected animal caps gain the capacity to form differentiated neurons when treated with 100 nM bexarotene (Fig. 4). Transcripts from these samples are compared to equivalent explants that are not exposed to bexarotene, and DEGs with a more than fivefold change and an adjusted *P*-value of less than 1.10^−5^ are selected for further analysis. This approach identifies 412 downregulated genes, of which 294 are currently annotated as protein-coding genes, and 968 upregulated genes, of which 892 are protein coding (Table S4). The reduction in numbers relates to the incomplete annotation of the *X. laevis* genome and the presence of non-protein-coding transcripts.

Considering the 294 downregulated genes (Fig. 6A), 46 are found in the AnimalTFDB4 database of transcription factors (Table S5), and 47 are recognised as factors that regulate RNA polII activity in the GO-term overrepresentation analysis of all downregulated genes (Fig. S2). Using just the genes related to transcription identifies GO terms related to head, brain and forebrain development (Fig. 6B) including *arx* (Seufert et al., 2005), *fezf1* (Shimizu and Hibi, 2009), *fezf2* (Zhang et al., 2014), *foxd1* (Polevoy et al., 2017), *hesx1* (Ermakova et al., 2007; Martynova et al., 2004), *otx1/2* (Andreazzoli et al., 1997) and *zic1* (Merzdorf and Forecki, 2018), which contribute to anterior neural development; *ag1* (Tereshina et al., 2019), involved in cement gland and anterior neural fate determination; *rax1*, involved in retinal and anterior neural development (Andreazzoli et al., 1999); and *sall2*, which promotes anterior neural development by attenuating Wnt signalling (Onai et al., 2004) (genes labelled in Fig. 6A). *sox21* is also downregulated and is a gene of interest, as its expression is required at a low level for primary neuron formation, whilst high levels repress neuronal differentiation (Whittington et al., 2015). Enhanced RXRβ-bexarotene signalling therefore diminishes the ability of the explants to form neural ectoderm with an anterior identity.

Downregulated genes involved in cell signalling unsurprisingly include *bmp4* and *7* and the receptor *bmpr1b* (*alk6*) in addition to *agr2*, a cement-gland-secreted signal (Tereshina et al., 2019). In addition to transcription factors and signalling molecules, three other genes feature strongly in the volcano plot. *lrp2* is involved in neural tube closure (Kowalczyk et al., 2021), whilst *bcas1* expression, in mice, is a marker for a subset of oligodendrocytes (Fard et al., 2017) and *gria2* encodes a synaptic glutamate [α-amino-3-hydroxy-5-methyl-4-isoxazolepropionic acid (AMPA)] receptor (Cotton and Partin, 2000).

To refine the analysis, we identified 178 genes that are initially activated in response to the injection of noggin and RXRβ mRNAs alone but downregulated at least fivefold when comparable injected explants are treated with bexarotene (Fig. 6C; Table S6). GO-term analysis of the 144 annotated genes in this list identifies terms relating to forebrain and brain development as the most overrepresented, whilst terms relating to RNA polII transcription have the most significant *P*-values (Fig. 6D). Consequently, many transcription factors that promote anterior neural development and neural precursor proliferation that are upregulated in response to noggin/RXRβ mRNA injection are subsequently downregulated when RXRβ-bexarotene signalling is activated.

### Genes whose enhanced expression is maintained in response to Noggin/RXRβ mRNA with or without bexarotene include those that balance the processes of proliferation and differentiation

There are 567 genes upregulated in response to Noggin/RXRβ mRNA injection whose expression is then relatively maintained (less than fivefold change up or down) following bexarotene treatment (Fig. 6C; Table S7), of which 396 are annotated. GO-term analysis (Fig. S3) identifies genes with a role in CNS development as the most overrepresented and those involved in transcription as the largest set. These include the genes *sox2*, *3* and *4*, which generally maintain the proliferation of neural precursors, suggesting a need to balance the process of neuronal differentiation and the continued proliferation of precursors. *sox11* (Singleton et al., 2024) is also maintained and, along with *sox2*, has been identified as a pioneer factor that can open chromatin to subsequently enable the activation of genes involved in neuronal differentiation (Dodonova et al., 2020). Consistent with these functions is the maintenance of expression of the gene encoding the RNA-binding protein Msi1 that regulates progression from the proliferative state (MacNicol et al., 2015). Also included are the REST corepressor genes *rcor1* and *2* that have been implicated in balancing proliferation and neuronal differentiation (Lakowski et al., 2006; Monaghan et al., 2017; Zeng et al., 2010) and the gene *marcks* that encodes a protein kinase C substrate involved in neural tube closure (El Amri et al., 2024), a function shared by genes in the GO-term actin filament-based process including *shroom2* and *3* (Chu et al., 2013).

### Genes that are upregulated when Noggin-/RXRβ-mRNA-injected animal caps are treated with bexarotene promote neuronal differentiation and contribute to AP identity

There are 892 genes that meet the criteria (>5× change in expression, *P*_adj_<10^−5^) for upregulated DEGs in Noggin-/RXRβ-mRNA-injected explants subject to 100 nM bexarotene (Fig. 7A; Table S4). Biological GO-term analysis identifies terms associated with a range of processes, notably AP patterning, canonical Wnt signalling and CNS development (Fig. S4). Comparison of the 892 upregulated genes to the catalogue of 1060 genes in the *X. tropicalis* database TFDB4 generates an overlap of 110 genes (a *z*-score of 9.7) (Table S5). Included in this list is the proneural gene *neurog2* (Ma et al., 1996), but not *neurod*, a differentiation factor that, in whole embryos, is expressed later, after the gastrula stages (Lee et al., 1995). The gene *myt1* (Bellefroid et al., 1996), activated by *neurog2*, is necessary for neuronal differentiation and is also elevated in expression in response to enhanced RXRβ-bexarotene signalling as is *mxi1*, implicated in the initiation of neurogenesis (Klisch et al., 2006).

Transcription factors that contribute to the establishment of an AP axis are also upregulated, including the *cdx1/2/4* genes, several hox genes and retinoid receptors (labelled in Fig. 7A). The previously identified indicator genes (Fig. 2) of standard retinoid signalling are also activated in response to RXRβ-bexarotene signalling. In the case of *rara*, the characterised RARE in the first intron specifically activates transcription of the *rara2* isoform, and this is seen in the RNA-seq output from the bexarotene-treated embryos (Fig. 7B).

A range of genes that relate to intercellular signals are found amongst the upregulated genes (labelled in Fig. 7C) and PANTHER Pathways identifies Wnt signalling (28 genes) including *wnt3a*, *4*, *5a/b, 7a/b*, *9* and *11b* and frizzleds *fzd1*, *6* and *10* (Fig. 7D). In addition, several genes whose proteins antagonise Wnt signalling, including *sall2* (Onai et al., 2004), are downregulated (labelled in Fig. 7A,C). The canonical Wnt pathway remains a major identified pathway when the DEG selection stringency is increased or decreased; however, non-canonical signalling is not identified as a significant pathway amongst upregulated genes at any stringency tested (Fig. S5). The canonical Wnt signalling pathway is implicated in imparting posterior character to neural-ectodermal cells (Kiecker and Niehrs, 2001; Nordström et al., 2002). PANTHER Pathways also identifies FGF signalling (13 genes) including *fgf3*, *8* and *20* and *fgfr1* and *3*. The timing and location of the expression of *fgf3* and *8* in normal development indicate a role in the formation of neurons in the caudal neural plate (Polevoy et al., 2019).

Although this paper concentrates on neurogenesis, other developmental events may be taking place in the explant. Genes involved in determining the left-right axis (Hamada and Tam, 2020) are also upregulated by bexarotene signalling including *nodal*, *lefty*, *dand5*, *pitx2*, *fgf8* and *foxh1*. In addition, the three upregulated genes associated with cilia formation and function, *rfx4*, *shroom3* and *ttc21b* (Fig. 5A), are each associated with left-right patterning (Bonnafe et al., 2004; Deng et al., 2026; Tariq et al., 2011). It is not apparent, though, whether a left-right axis is established in the explants. Indeed, nodal is one of the most significantly upregulated genes and has been implicated as a contributor to neural induction, in addition to its pivotal role in left-right patterning (Le Petillon et al., 2017).

**Fig. 8.**
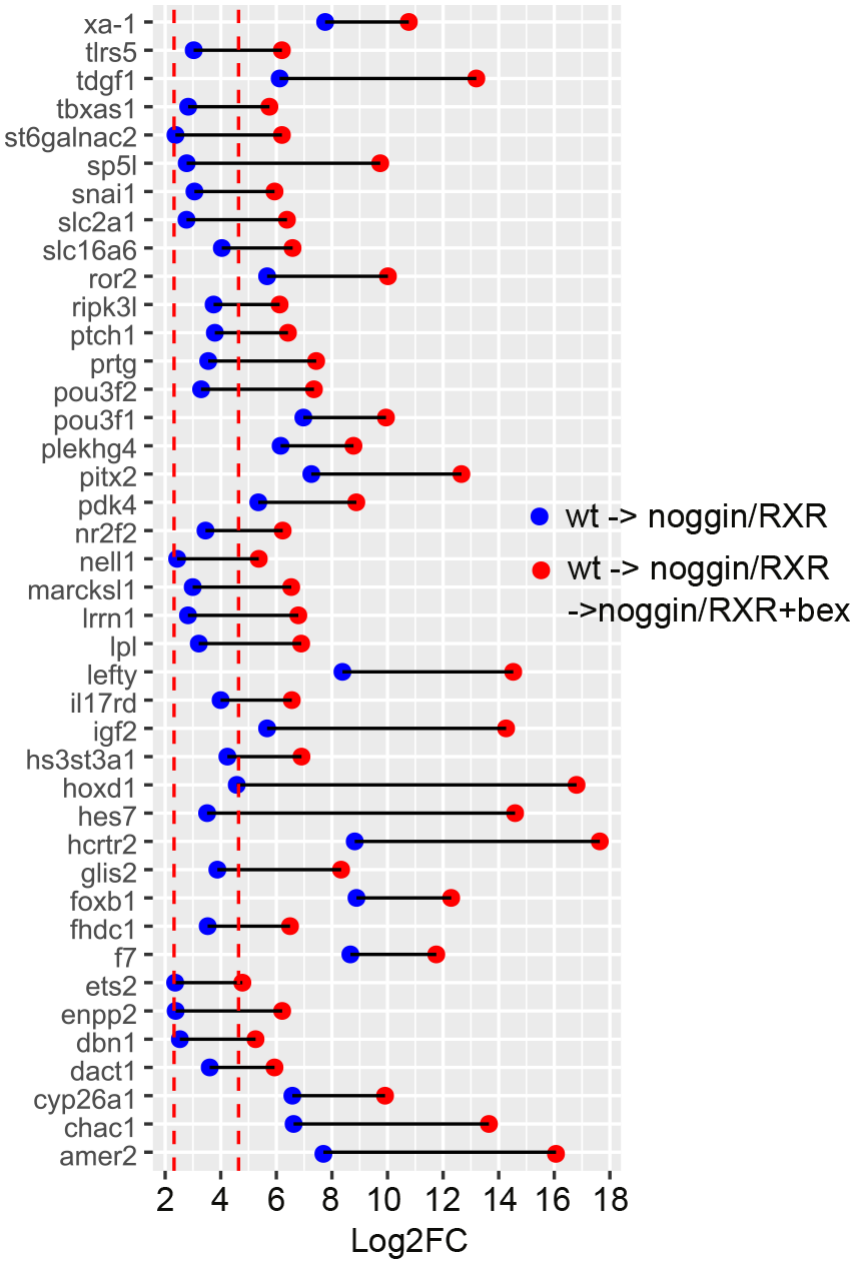
**Forty-one genes are upregulated at least fivefold in response to Noggin and RXRβ mRNA injection and a further fivefold if injected animal caps are also treated with bexarotene.** Cleveland plot with the 41 doubly upregulated genes identified along the *y*-axis. The *x*-axis represents the value of the fold change in expression in response to injection of just the two mRNAs (blue dot) and the composite response to mRNA injection and the addition of 100 nM bexarotene (red dot). The first dotted red line represents a fivefold change and the second a 25-fold change on the log2 fold-change *x*-axis compared to untreated animal cap explants.

A final subset of DEGs comprises 41 genes upregulated in response to Noggin/RXRβ mRNA injection and further upregulated when these explants are subject to bexarotene (Fig. 8; Table S8). Several genes relating to key aspects of neuron formation are found in the list, including *hoxd1*, a direct target of both retinoid and Wnt signalling involved in the establishment of AP pattern; *glis2*, a gene activated by Neurog2 that plays a role in activating *xmyt1* (Lamar et al., 2001); and *chac1*, which, amongst other activities, interacts with Delta-Notch signalling to promote neuron formation (Sun et al., 2024). The activation of these genes in Noggin-/RXRβ-mRNA-injected explants in the absence of bexarotene suggests that this treatment, in addition to promoting anterior and proliferative states, also activates genes that will subsequently contribute to neuronal differentiation.

## DISCUSSION

The formation of a functional, vertebrate nervous system is a remarkable event in developmental biology even when, as in the case of the *X. laevis* embryonic nervous system, it comprises less than 2,000 neurons, has a limited number of synapses and has only a small number of cell types. Nevertheless, these neurons form active, regulated circuits that control swimming movements and basic behaviours (Roberts et al., 2010). They also have merit as a simple tool to shed light on the fundamental process of neuron formation in more complex systems (Exner and Willsey, 2021). *X. laevis* embryos are easy to inject with synthetic mRNA, and the isolation of animal cap explants is a standard procedure. Neuronal differentiation occurs within 2 days and is readily detected with immunohistochemical or molecular approaches, providing a rapid, high-throughput method to identify genes and bioactive molecules that promote neuron development.

Despite the apparent simplicity of the animal cap explant, its response to the inhibition of BMP signalling and the activation of RXR-bexarotene signalling results in changes in the expression of several hundred genes. Collecting samples within 4 h of treatment identifies the early steps confirmed by the inclusion of the neurogenic gene, *neurog2*, but not the later-expressed differentiation gene, *neurod*, in the list of DEGs. Indeed, we concentrate on DEGs that are involved in transcription or intercellular signalling, rather than on the genes that mediate the later function of neurons such as axon guidance, synapse formation, and neurotransmission.

The formation of EPNs first requires ectodermal cells to be diverted to a neural fate in a process that can be divided into four steps: the inhibition of BMP signalling, the consequent suppression of mucociliary epidermal development, the downregulation of inhibitors of neural development such as *ventx2* and the promotion of a proliferative neural precursor state. At this point, cells do not progress to a neuronal fate; however, several genes are expressed that will promote neuronal development in conjunction with subsequently expressed genes. One example is *glis2*, whose protein will interact with Myt1 to promote neuronal differentiation (Lamar et al., 2001).

Retinoid signalling promotes neuronal differentiation in Noggin-neuralised ectodermal cells (Papalopulu and Kintner, 1996; Sharpe and Goldstone, 2000b). The specificity of retinoid signalling in this process is broad, being mediated by both RARα and RARγ as heterodimers with RXRβ along with the ligand *at*RA (Fig. 1). Here, we show that the addition of bexarotene to Noggin-neuralised ectodermal cells programmed to express RXRβ activates many of the genes on the established pathway to neuronal differentiation. Bexarotene is required at the beginning of gastrulation, indicating a role in the early stages of neuron formation, as seen previously for *R*AR/RXR-*at*RA-mediated neurogenesis (Sharpe and Goldstone, 2000a). In explants, RXRβ-bexarotene signalling upregulates *rara* expression (Fig. 7A), which may then facilitate the activation of the standard retinoid signalling pathway of neuronal development. Consequently, RXR-bexarotene signalling leads to the expression of genes including *neurog2*, *myt1*, *mxi1* and components of the Delta-Notch signalling pathway that selects those cells that will terminally differentiate into EPNs (Chitnis and Kintner, 1995).

The ability to differentiate EPNs is linked to the processes that determine the AP identity of the neural-ectodermal cells of the embryo. Standard retinoid signalling regulates the expression of *cdx1/2/4* and a range of *hox* genes (Gould et al., 1998; Houle et al., 2000; Keenan et al., 2006; Schilling and Knight, 2001), and these genes are upregulated in the Noggin/RXRβ-bexarotene explants. These explants also upregulate many of the components of the canonical Wnt signalling pathway that is implicated in imparting posterior character to neural-ectodermal cells (Kiecker and Niehrs, 2001; Nordström et al., 2002). Since these observations relate to the explants, it would be interesting to determine how the expression of posterior patterning and Wnt signalling genes relates to individual cells within the subpopulations of neural precursor and non-neural cells. Together, these results suggest that bexarotene-mediated signalling promotes neuron development in a manner closely related to that used by standard retinoid signalling via RAR/RXR-*at*RA that integrates positional signals and those directly related to neurogenesis.

**Fig. 9.**
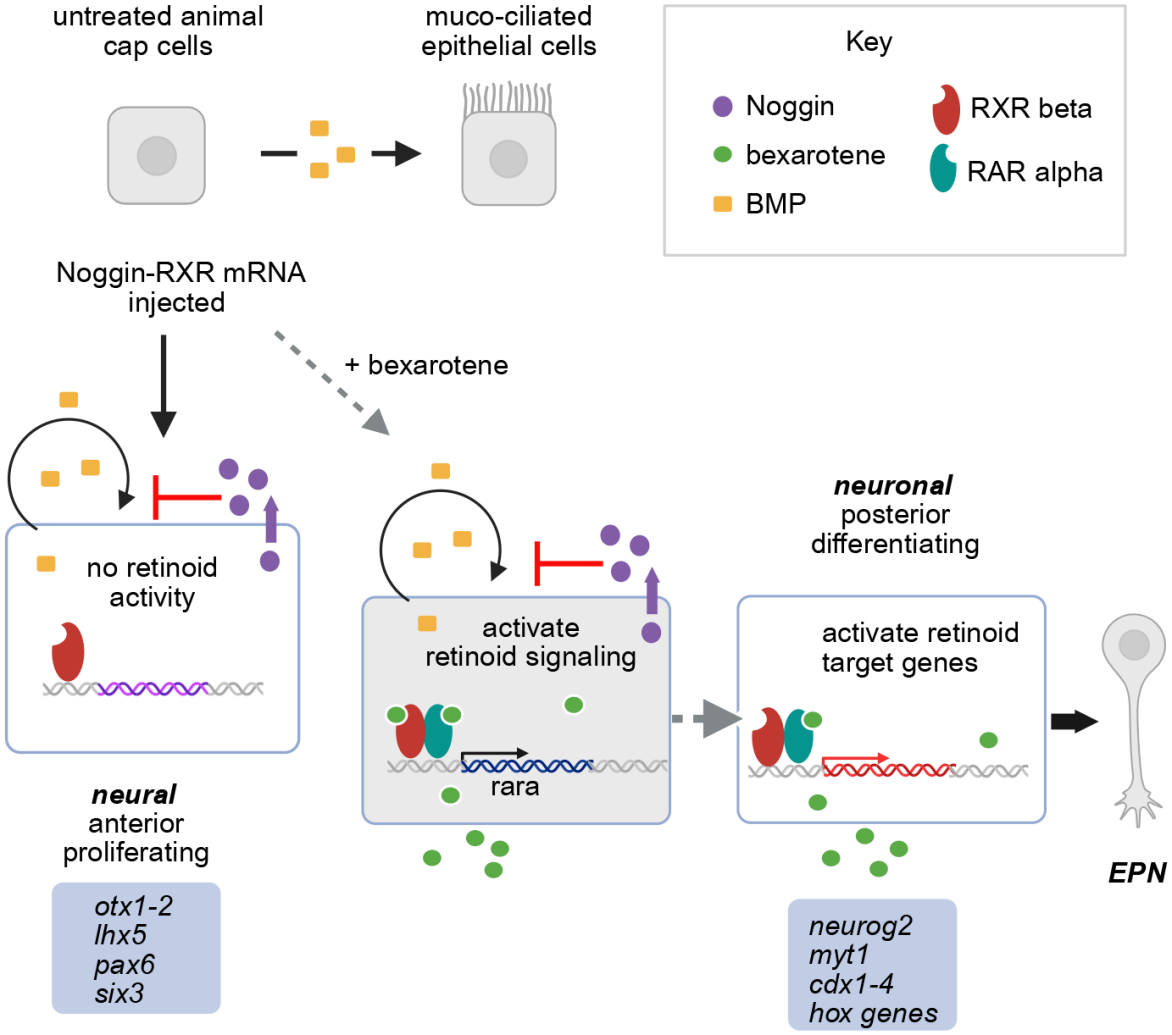
**The proposed model for the promotion of neuronal differentiation by the activation of RXRβ-bexarotene signalling in neuralised ectodermal cells.** The elevated levels of *rara* mRNA following the injection of noggin and RXRβ mRNA and treatment with bexarotene suggest a model in which bexarotene activates the standard RAR/RXR-*at*RA pathway, promoting the differentiation of EPNs within an explant that demonstrates posterior character. Representative genes are shown in the blue boxes. Created in BioRender by Sharpe, C. (2026). https://BioRender.com/t6hddb7. This figure was sublicensed under CC-BY 4.0 terms.

Characterised indicator genes of standard retinoid signalling such as *cyp26a1* (Zhang et al., 2014) and *hoxb4* (Gould et al., 1998) (Fig. 1) respond through DR5 RAREs that would not be expected to have a strong affinity for bexarotene-liganded RXR. The specificity of bexarotene, however, has been questioned, and it is likely that bexarotene also interacts, if less strongly, with RAR/RXR heterodimers (Isigkeit et al., 2024). This is further supported by the upregulation of the *rara* gene, which has a defined DR5-RARE driving its P2 promoter (Fig. 7B). Although some RXR agonists activate RAR/RXR signalling through the enhanced production of *at*RA (Melo et al., 2023), we do not see an increase in the expression of genes encoding retinaldehyde dehydrogenase activity in response to bexarotene. This paper uses a gain-of-function analysis to look at RXRβ-bexarotene activity. An alternative would be to knock down the expression of RXRβ and ask whether bexarotene can still promote the formation of additional neurons. This would be likely, however, to cause additional effects as heterodimerisation with an RXR protein is required for the activity of many of the type II nuclear receptors. One hypothetical model for the ability of RXR-bexarotene signalling to promote neuronal development in *X. laevis* is that RXRβ protein, following injection of its mRNA, together with its high-affinity ligand, bexarotene, and residual RARα protein, upregulates transcription from the *rara* gene, which then uses newly made RARα protein in standard RAR-RXR retinoid signalling, maintaining bexarotene as the activating ligand (Fig. 9).

### Limitations

In this paper, we identify DEGs in response to RXR-bexarotene signalling in *X. laevis* embryos. We have not independently tested the identified genes and rely on stringent selection criteria for the DEGs (a minimum of a fivefold change in expression with *P*_adj_<10^−5^). Many of the RXRβ-bexarotene-responsive genes have previously been confirmed as standard retinoid-responsive genes, though we have not compared the current results to an RNA-seq analysis of RAR/RXR-*at*RA-treated Noggin-neuralised animal caps. Future steps would include examining whether the expression of *rara* precedes that of genes such as *neurog2* in the RXR-bexarotene-treated animal caps and identifying the promoters and genes to which RXRβ binds, when activated by bexarotene, to distinguish those using RXREs from those that use RAREs.

### Conclusion

Bexarotene activates the expression of RXR-responsive genes *abca1* and *apoe* whose proteins are involved in clearing amyloid-β in the brain (Corona et al., 2016; Lefterov et al., 2015), though it remains uncertain whether bexarotene can reduce pathogenic levels of plaque in mouse models of Alzheimer's disease (Landreth et al., 2013; Tousi, 2015) and has been ineffective as a therapy in clinical trials (Cummings et al., 2016). Bexarotene, however, promotes aspects of neurogenesis to regenerate lost neuronal connectivity in these models (Mounier et al., 2015; Saibro-Girardi et al., 2024), generating interest in understanding how bexarotene influences neuronal development. Although neurogenesis in the adult mammalian brain is likely to integrate a range of regulatory signals, we demonstrate the ability of bexarotene to promote neuron formation using *X. laevis* ectodermal explants as a rapid and accessible tool to examine the role of nuclear receptors in neurogenesis. RXR-bexarotene-driven neuronal differentiation in this system involves changes in gene expression that match extensively to those seen when RAR/RXR-*at*RA signalling promotes neuron formation in *Xenopus* embryos.

## MATERIALS AND METHODS

### Generation and maintenance of *Xenopus* embryos

*X. laevis* embryos provided by the European *Xenopus* Resource Centre (EXRC) (https://xenopusresource.org/) were maintained in 1× modified Barth's solution (MBS) (Gurdon, 1977) and staged by reference to a normal table of development (Nieuwkoop and Faber, 1958). Embryos were injected at the two-cell stage in 3% Ficoll, 1× MBS. Where appropriate, embryos and animal caps were grown from the gastrula stage in 100 nM bexarotene (Sigma-Aldrich) diluted 1:1000 into 0.1× MBS (1× MBS for animal caps) from a 10^−4^ M stock in ethanol. Bexarotene is typically used at concentrations between 30 nM (Mengeling et al., 2018) and 1 μM (Saibro-Girardi et al., 2024), and *Xenopus* embryos survive to tailbud stages equally well in a 1:1000 dilution of absolute ethanol (untreated control) or 100 nM bexarotene. Embryos were grown in 100 nM *at*RA diluted 1:1000 from a 10^−4^ M stock in DMSO, and untreated controls were grown in a 1:1000 dilution of DMSO.

Typically, embryos were injected with 125-250 pg mRNA (Guille, 1999). Synthetic mRNAs were generated from vectors replacing the noncoding regions of mRNA with those from *Xenopus* beta globin to ensure efficient translation. In some cases, embryos were co-injected with 125 pg β-galactosidase mRNA as a lineage tracer, and X-gal (Sigma-Aldrich) was used as a chromogenic substrate for β-galactosidase enzyme expression. Following injection, embryos were maintained in 0.1× MBS until the required stage and then either fixed in MEMFA fixative (Guille, 1999) and stored in methanol for immunohistochemistry, frozen at −80°C awaiting RNA extraction, or processed for animal cap preparation. Noggin and RXRβ clones used are available from the EXRC.

Adult *X. laevis* were housed within the EXRC, University of Portsmouth. All work was conducted in accordance with the Home Office Code of Practice under PPL PP1960991 following approval from the University of Portsmouth's Animal Welfare and Ethical Review Body.

### Generation and maintenance of animal caps

The vitelline membrane was removed from embryos between NF stages 9 and 10, and animal caps were dissected in 1× MBS. At least six caps per treatment were maintained as pairs with opposing inner faces. The outer ectodermal cell coat reforms more easily around a pair than an individual animal cap, enhancing explant survival. The explants are either fixed, as above, for immunohistochemistry when control embryos reached the late neurula stage (Figs 1,4) or frozen at −80°C at the late neurula stage (Figs 1,4) or after 4 h of culture at 21°C for RNA-seq analysis and then used for subsequent RNA extraction.

### Extraction and preparation of embryo RNA

Embryos were typically frozen at −80°C in groups of four or groups of three paired animal caps and nucleic acids recovered by homogenisation in 1× sodium chloride, EDTA, Tris-HCl, SDS (NETS) buffer followed by two rounds of extraction with phenol-chloroform and ethanol precipitation (Guille, 1999). DNA was removed by treatment with DNase 1, and the RNA was recovered by phenol-chloroform extraction and ethanol precipitation. Samples were quantified using a NanoDrop spectrophotometer and checked by electrophoresis on a 1.2% agarose gel in 1× Tris-HCl, Borate, EDTA (TBE) buffer.

### RT-PCR analysis

Extraction typically generated around 5 μg of total RNA per embryo, and approximately 10 μg was used in 20 μl reverse transcriptase reactions (PCRBio UltraScript cDNA Synthesis Kit). Semi-quantitative PCR was performed using 1 μl of cDNA and appropriate primers (Table 1) for between 28 and 32 cycles, and products were resolved on 1.2% agarose gels in 1× TBE buffer. Results shown are typical gels from at least two repeats of the reactions.

**Table 1.** List of primers

Gene	Sequence 5′-3′ forward	Sequence 5′-3′ reverse
*cdx4*	GGATCACCGAGGGAGGAATG	TAAGAGCGCTGGGTGAGTTGG
*cyp26a1*	GCTGCCACGTCCCTCACCTCTT	GCCGATGCAGCACCTCACTCCA
*hoxb4*	TGTGGACCCCAAGTTTCCGCC	CTTGTGCTCTGAGGACTTGAG
*Ncoa2*	AATCCTGCGTACCAGTCA	GTAATCTTTCGGGTCAGC
*Odc*	CAGCTAGCTGTGGTGTGG	CAACATGGAAACTCACACC
*wnt3a*	CTGAGGCCCAAATACACATTCT	GACCGCAACATAAAAGATCACA
*Rxrb.L* (exon 1)	GGGCTGGAAGCTGCGGGTCCG	
*Rxrb.L* (exon 2)	ACCAAGATGATGGTATTTCCG	
*Rxrb.S*	GCTGCTGGGTCCGGTGCGAGG	
*Rxrb* (S and L)		CTCCTGGCATTCCTGGAACT

### *In situ* hybridisation

For *in situ* hybridisation, animal caps fixed in MEMFA and stored in methanol were rehydrated in 1× PBS and subjected to a standard *in situ* hybridisation protocol (Guille, 1999) using a digoxygenin-labelled RNA probe antisense to neural-specific tubulin beta 2B class IIb (*tubb2b*) transcripts. Following washing, the embryos were stained using alkaline-phosphatase-labelled anti-digoxygenin Fab fragments and the chromogenic alkaline phosphatase substrate Roche BM-purple (Sigma-Aldrich).

### Immunohistochemistry

Fixed embryos stored in methanol were rehydrated in 1× PBS. The anti-Islet1 primary monoclonal antibody 39.4D5, developed by Jessell and Brenner-Morton, was obtained from the Developmental Studies Hybridoma Bank, created by the National Institute of Child Health and Human Development of the National Institutes of Health and maintained at the University of Iowa, Department of Biology, Iowa City, IA 52242, USA. In *X. laevis*, the antibody recognises an epitope that identifies predominantly the nuclei of Rohan-Beard embryonic primary sensory neurons*,* permitting quantification of this cell type. The primary mouse IgM monoclonal antibody VC1.1 recognises an epitope on human natural killer cells (anti-HNK-1) (Sigma-Aldrich). However, in *X. laevis*, it primarily recognises an epitope associated with NCAM and identifies axons and the Golgi apparatus of EPNs and some neural crest cells. In both cases, the primary antibody was used at a dilution of 1:250 and the appropriate horseradish peroxidase (HRPO)-linked secondary antibody at 1:500. This is a goat anti-mouse IgG secondary for 39.4D5 and a goat anti-mouse IgM secondary for VC1.1. Binding was visualised using a metal-enhanced 3,3′-diaminobenzidine substrate (Thermo Fisher Scientific), and embryos were rendered transparent in Murray's clear for photography on a Zeiss Axio Zoom.V16 Stereomicroscope.

### RNA-seq analysis

Three pairs of uninjected animal caps were used to generate one untreated sample, and this was repeated two more times to generate three replicates. The same process was performed to generate three replicates, each containing three pairs of animal caps for the samples injected with noggin and RXRβ mRNAs and noggin and RXRβ mRNAs plus bexarotene. The nine samples were subject to RNA sequencing by Novogene. RNA-seq quality control data are in Table S1.

Sequence reads were mapped against the *X. laevis* genome (v10). Unannotated loci were manually checked against current data in Xenbase (https://www.xenbase.org/xenbase/), updated where appropriate or removed from consideration if they remained unannotated. Due to the allotetraploid nature of the *X. laevis* genome, where two alloalleles (from the L and S subgenomes) are identified as differentially expressed, only the most significant alloallele, as determined by the fold change in expression and the resulting adjusted *P*-value, is included in volcano plots and GO-term overrepresentation analysis.

DEGs were identified using DESeq2 available via Bioconductor (Love et al., 2014) and defined by a false discovery rate of ≤0.05. The least stringent criteria used to select DEGs of interest required a minimum fivefold change in expression and an adjusted *P*-value of less than 10^−5^, thereby prioritising strongly regulated genes. Volcano plots were constructed in R/ggplot2. Selected DEG lists were compared with the CiliaCarta Gold gene list (Van Dam et al., 2019) or with the list of genes known to influence transcription (AnimalTFDB4 *X. tropicalis*) (Shen et al., 2023) using a Venn diagram, and the *z*-score of the overlap was determined assuming that the *X. laevis* genome consists of 19,500 genes (ignoring alloalleles). A *z*-score greater than 4 is deemed of interest. Selected DEG lists were subject to GO-term overrepresentation analysis using the AMIGO2 site driven by the PANTHER Classification System (https://amigo.geneontology.org/amigo) (Carbon et al., 2009) using the *X. tropicalis* genome list for comparison and Fisher's exact test and Bonferroni correction for multiple testing. For short lists, GO-term complete lists were used, and for longer lists, the GO-Slim comparisons were used. The PANTHER Pathways process in AMIGO2 identified relevant signalling pathways.

## Supplementary Material



10.1242/biolopen.062675_sup1 Supplementary information

Tables S2-8.
