# Supplementary material for: Signalling through RXRβ and its agonist, bexarotene, promotes neuron formation in Xenopus laevis embryos
Source: Biol Open. 2026 Jul 16;15(7):bio062675. doi: 10.1242/bio.062675 (PMC13446568; doi:10.1242/bio.062675)
Supplement: Supplementary file 1 [file biolopen-15-062675-s1.pdf]

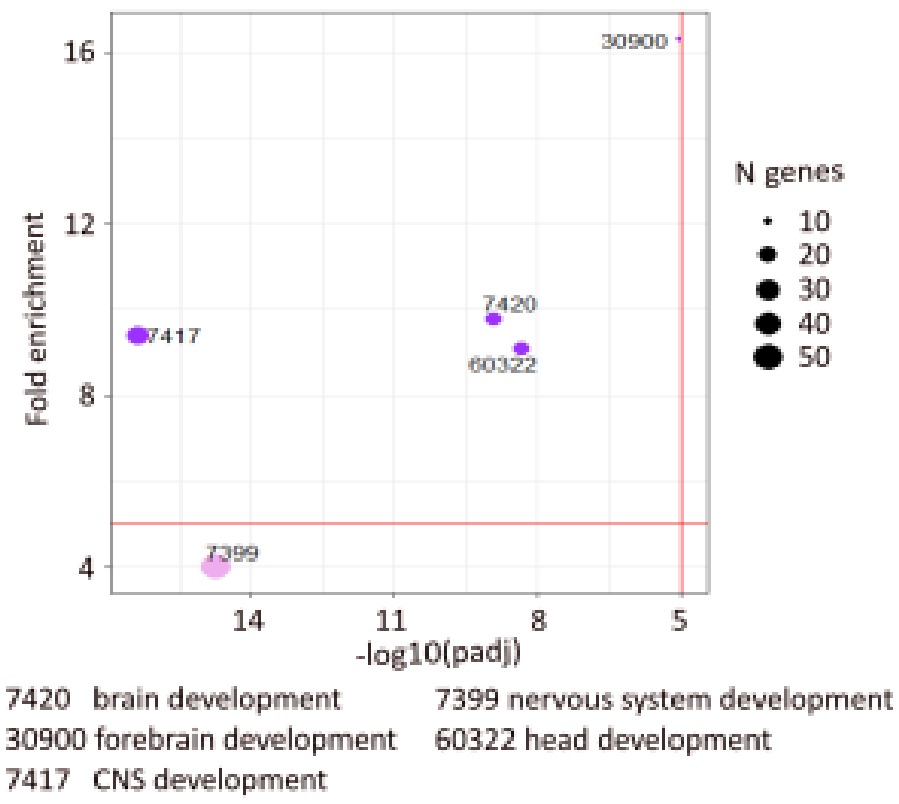

Fig. S1. GO term over-representation analysis of upregulated differentially expressed genes in Noggin and RXRβ mRNA injected compared to untreated animal caps.

The over-represented terms each relate to brain or neural development. The red lines mark the imposed limits of significance.

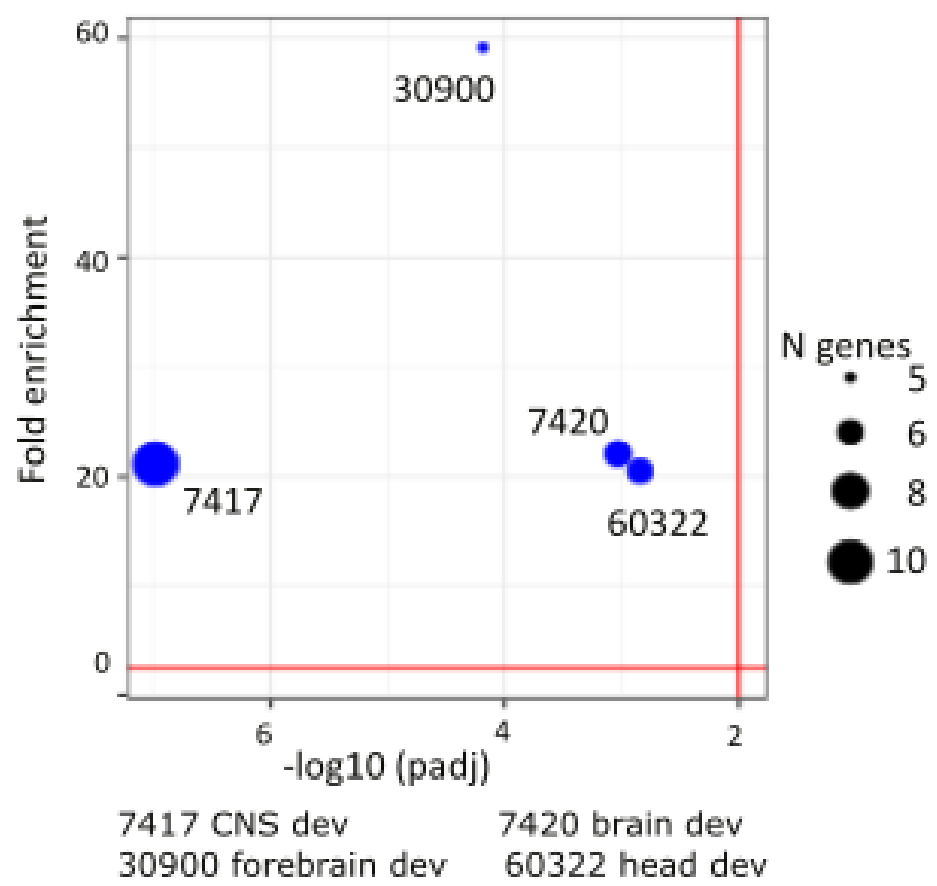

Fig. S2. GO term over-representation analysis of downregulated differentially expressed genes in Noggin and RXRβ mRNA injected animal caps with bexarotene compared to similarly treated animal caps in the absence of bexarotene.

The over-represented terms each relate to brain or neural development. The red lines mark the imposed limits of significance.

Biology Open • Supplementary information

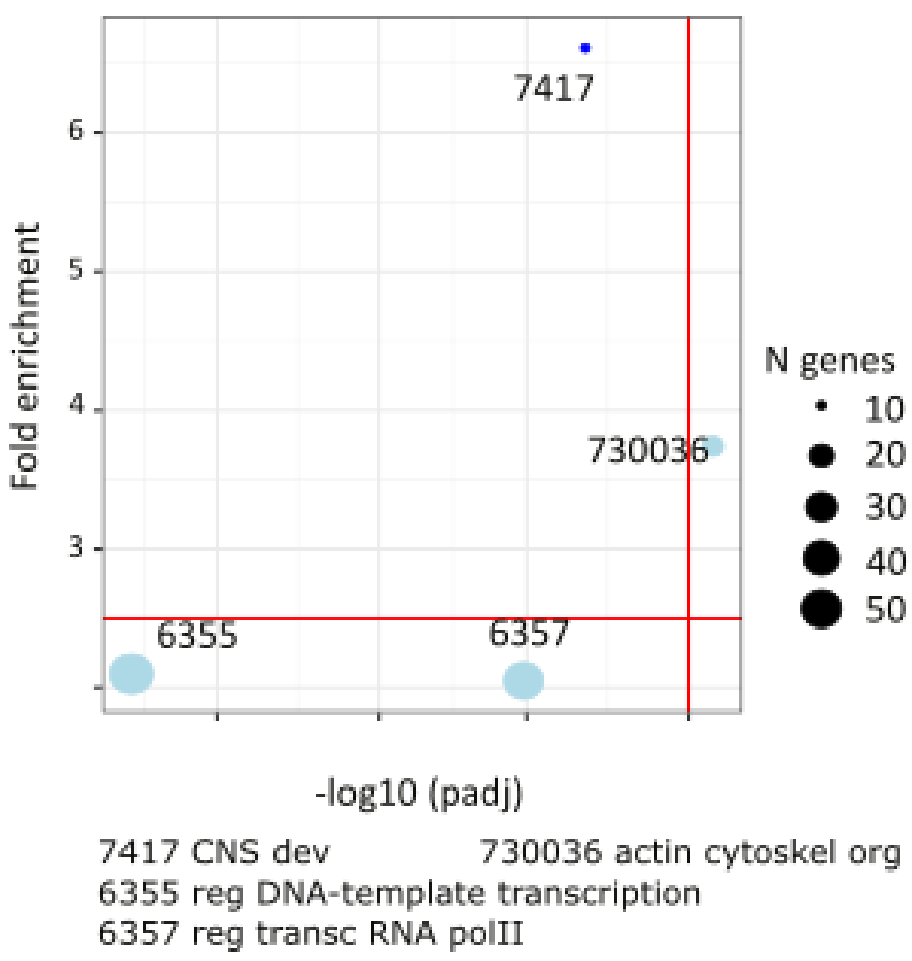

Fig. S3. GO term over-representation analysis of genes upregulated in Noggin and RXRβ mRNA injected animal caps whose expression is maintained when bexarotene is added to similarly treated animal caps.

The over-represented terms relate primarily to CNS development. The red lines mark the imposed limits of significance.

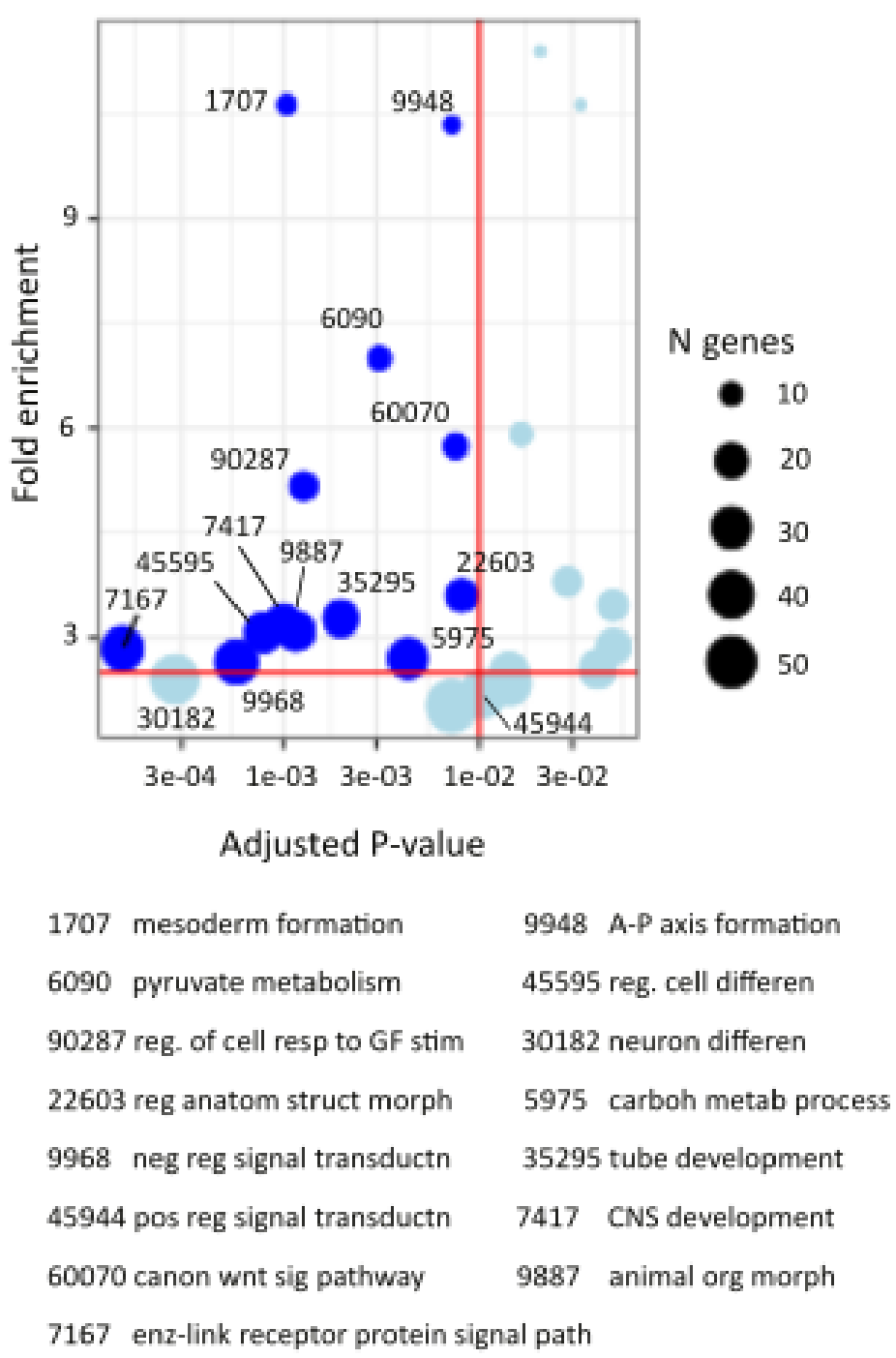

Fig. S4. GO term over-representation analysis of genes upregulated in Noggin and RXRβ mRNA injected animal caps given bexarotene compared similarly treated animal caps without bexarotene.

A range of GO-terms is identified including AP axis formation, neural development and the canonical Wnt signaling pathway. The red lines mark the imposed limits of significance.

Biology Open • Supplementary information

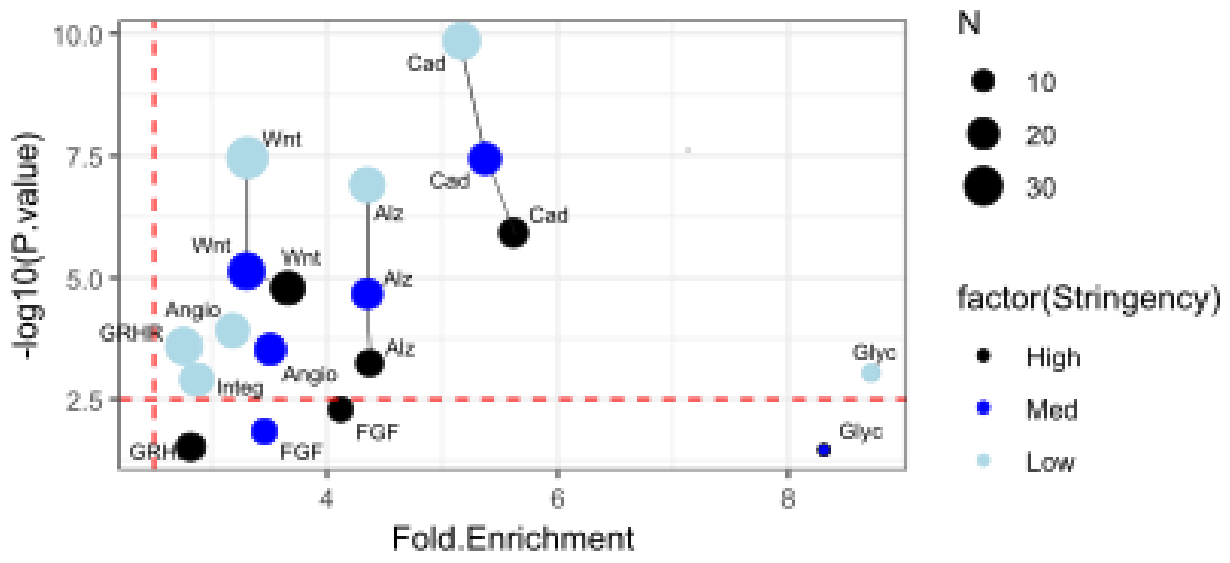

Fig. S5. Panther pathway analysis of differentially expressed genes upregulated in Noggin/RXRβ mRNA injected animal caps given bexarotene compared to similar caps in the absence of bexarotene

The analysis was performed at three stringencies: Low (2.5x fold change/<10^{-4} padj), medium (5x fold change/<10^{-5} padj) and high (7.5x fold change/<10^{-6} padj). In each case the predominant pathways are canonical Wnt signalling, Cadherin signalling and intercellular signals associated with Alzheimer's disease. These three pathways have an extensive overlap of genes.

Biology Open • Supplementary information

Table S1. Summary of RNAseq quality control and alignment metrics

Sample	Condition	Clean Reads (millions)	Q30 (%)	GC (%)	Total Mapped (%)	Unique Mapped (%)	Properly Paired (%)
ACWT1	WT	42.4	97.97	44.24	91.13	87.48	80.29
ACWT2	WT	50.5	97.92	44.48	91.22	87.70	81.16
ACWT3	WT	42.6	97.40	43.19	88.92	85.64	77.07
NGRX1	NGR	78.2	97.61	41.47	85.61	83.13	71.80
NGRX2	NGR	41.4	97.70	43.24	89.97	86.88	78.19
NGRX3	NGR	46.1	97.22	42.76	90.16	87.01	78.77
BEX1	+BEX	43.8	97.91	44.57	90.47	86.44	78.82
BEX2	+BEX	42.0	97.75	44.92	91.10	87.79	81.21
BEX3	+BEX	43.8	97.89	44.43	91.29	87.88	81.19

RNA-seq libraries generated ~41-78 million clean reads per sample, with high sequencing quality (Q30 >97%) and consistent GC content (41-45%). Alignment rates were high across all samples, with 85-91% of reads mapping to the reference genome and 83-88% mapping uniquely. Properly paired reads ranged from 72-81%, indicating robust library quality and consistent performance across biological replicates.

ACWT = wild type animal caps (untreated)

NGRX = animal caps from embryos injected with Noggin and RXRb mRNA.

BEX = animal caps from embryos injected with Noggin and RXRb mRNA grown in 100nM bexarotene.

Tables S2-8.

Available for download at
https://journals.biologists.com/bio/article-lookup/doi/10.1242/bio.062675#supplementary-data

Biology Open • Supplementary information
