# Supplementary material for: Signalling through RXRβ and its agonist, bexarotene, promotes neuron formation in Xenopus laevis embryos
Source: Biol Open. 2026 Jul 16;15(7):bio062675. doi: 10.1242/bio.062675 (PMC13446568; doi:10.1242/bio.062675)
Supplement: Supplementary file 2 [file biolopen-15-062675_review_history.pdf]

Signaling through RXRß and its agonist, bexarotene, promotes neuron formation in Xenopus laevis embryos

Daniela Lopes Cardoso, Marilena Loizou, Danai D. Fourla, Charles Woodman, Matthew J. Guille and Colin R. Sharpe
10.1242/bio.062675

Editor: Tristan Rodríguez

Review timeline

Original submission:	1 May 2026
Editorial decision:	8 May 2026
First revision received:	11 June 2026
Accepted:	15 June 2026

Original submission

First decision letter

MS ID#: bio.062675

MS Title: Signaling through RXRß and its agonist, bexarotene, promotes neuron formation in Xenopus laevis embryos

Authors: Daniela Lopes Cardoso; Marilena Loizou; Danai D. Fourla; Charles Woodman; Matthew J. Guille; Colin R. Sharpe

I have now reached a decision on the above manuscript.

The reviewer reports are shown at the bottom of this email.

As you will see, the reviewers raised a number of substantial criticisms that prevent me from accepting the paper at this stage.

They suggest, however, that a revised version might prove acceptable, if you can address their concerns. If you think that you can deal satisfactorily with the criticisms on revision, I would be pleased to see a revised manuscript. We would then return it to the reviewers.

At this stage, we also ask you to ensure your manuscript complies with our formatting guidelines. Provided you are able to fully address the referees' comments, we are positive about publication of your paper (we accept over 95% of revision submissions) and therefore hope you won't mind any extra work involved in reformatting your manuscript at this point.

Please upload both a 'clean' version of your Word file, along with a highlighted version clearly showing where you have made changes in the revised manuscript. Please avoid using 'Track changes' in Word files as these are lost in PDF conversion.

I should be grateful if you would also provide a point-by-point response detailing how you have dealt with the points raised by the reviewers in the 'Response to Reviewers' box. Please attend to all of the reviewers' comments. If you do not agree with any of their criticisms or suggestions please explain clearly why this is so.

© 2026. Published by The Company of Biologists under the terms of the Creative Commons Attribution License (https://creativecommons.org/licenses/by/4.0/).

Reviewer 1

Comments for the author

This manuscript investigates the ability of RXRß/bexarotene signalling to promote neuronal differentiation in BMP-inhibited Xenopus ectoderm and combines embryological manipulation with transcriptomic analysis to define the underlying transcriptional programme. The study identifies temporal requirements for RXR signalling, demonstrates induction of early primary neurons in animal cap explants, and characterises associated changes in gene expression linked to neural processes.

The animal cap explant assay is presented as a tractable screening platform for retinoid-related neurogenic ligands, and the transcriptomic dataset will be of value to the Xenopus and retinoid-signalling communities. The RNA-seq component substantially strengthens the manuscript by moving beyond a descriptive phenotype toward mechanistic interpretation. The manuscript contains several valuable observations that will likely be of interest to researchers studying neural induction, retinoid signalling, and Xenopus developmental systems. The limitations section is honest: it acknowledges the missing atRA RNA-seq dataset and the absence of functional validation.

However, the mechanistic conclusions frequently go beyond what the transcriptomic data alone can support, including the central claim that bexarotene and atRA treatments act through related pathways. The manuscript also has issues with clarity and paragraph density, and contains a noticeable number of typos and formatting errors.

Major general comments:

Throughout the text, mechanistic claims should either be experimentally strengthened with validation (proposed below) or softened in the writing. As it stands, the discussion's mechanistic claims are stronger than the data here support. Reframing the manuscript more explicitly as a developmental transcriptomic resource paper, rather than a definitive signalling mechanism paper, would resolve much of this without requiring new experiments.

The manuscript would also benefit substantially from paragraph restructuring and more careful punctuation. Comprehension is often hindered by dense paragraphs (>10 lines) that contain multiple concepts, interpretations, and gene catalogues. This causes reader fatigue and makes it difficult to identify what is central. One example is the section on page 13-14 starting "Considering these 294…", it would aid the reader if these type of sections are split into shorter paragraphs.

Major specific comments:

1. The "kick-start" model is speculative and not supported by the current data.

The proposed model in which RXRß/bexarotene signalling induces rara and thereby kick-starts canonical retinoid signalling is interesting but remains untested. The authors note increased rara expression but do not separate cause from correlation, and there is no direct evidence presented that RAR activity is required for the bexarotene response (if such evidence is in the manuscript, it should be highlighted more clearly).

The RNA-seq experiment never compares Noggin/RXRß + bexarotene with Noggin/RAR/RXR + atRA. Neuronal differentiation in Noggin + RXRß + bexarotene caps (Fig. 4) could in principle be driven by RXR partnerships other than RAR. The induction of Wnt and FGF pathway components (Fig. 7) could likewise indicate a parallel or alternative route to neurogenesis.

I would ask the authors either to soften this model in both text and figure, or to provide functional validation. For softening: Fig. 9 should be explicitly labelled "Proposed model/hypothesis" in the title, with an open question in the rara arrow (e.g. question mark/dashed arrow). For validation, possible options include inhibition of RAR signalling during RXRß/bexarotene treatment or an RNA-seq comparison of Noggin/RXRß + bexarotene with Noggin/RAR/RXR + atRA. I appreciate validating

© 2026. Published by The Company of Biologists under the terms of the Creative Commons Attribution License (https://creativecommons.org/licenses/by/4.0/).

is a substantial ask. Low-cost bulk RNA-seq (e.g Plasmidsaurus) would make this more tractable if the authors wish to pursue it, but reframing the claims is an acceptable alternative.

2. The rara2 isoform claim should not be relegated to "data not shown".

The specific upregulation of the rara2 isoform could be a piece of evidence for the kick-start model. A simple read-coverage plot from the RNA-seq across the rara locus, would substantially strengthen this claim without requiring new experiments.

3. HNK1 as a neuronal marker (Fig. 4).

HNK1 is also expressed by neural crest derivatives in Xenopus, so HNK1 positivity alone does not unambiguously identify neurons. I suggest the authors acknowledge that their assay shows HNK1-positive cells consistent with neuronal differentiation but requiring additional markers (or co-staining) to definitively confirm neuronal identity. It would also be helpful to briefly mention why animal caps were paired in this experiment. Additionally, the blue background of panels B-G makes the ßgal signal difficult to see in print; could the authors adjust contrast or LUTs to improve ßgal visibility?

4. Cell-type resolution in the bexarotene response (Fig. 7).

Bulk RNA-seq cannot determine whether the posterior patterning and Wnt-related transcriptional changes occur within neural precursor cells specifically, or in non-neural subpopulations of the explants. It also cannot resolve whether cdx/hox and Wnt induction co-occur in the same cells. Without single-cell or spatial validation by ISH, the attribution of posterior character specifically to the neurogenic lineage remains an inference, and this should be acknowledged in the text.

5. Sensitivity of the canonical Wnt enrichment to the DEG cut-off.

The DEG analysis uses a stringent cut-off and canonical Wnt is then highlighted via GO/pathway over-representation. This inference may be sensitive to the chosen threshold and could miss more modest but coordinated changes. I suggest the authors repeat the over-representation analysis across multiple DEG cut-offs and summarise whether canonical Wnt enrichment persists (confirming it is not an artefact of stringency) across the three replicates. It would also be interesting to know whether non-canonical Wnt components show coordinated changes across the three replicates at lower threshold.

6. Bexarotene concentration and specificity.

All bexarotene experiments use a single 100 nM concentration, without dose-response characterisation, receptor-selective antagonist controls, or assessment of off-target actions. The authors note in both introduction and discussion that bexarotene specificity has been questioned. I thus wonder why this is the dose chosen by the authors. This could be addressed through text edits. Perhaps noting that the chosen concentration is consistent with the mammalian bexarotene literature already cited (if so), and acknowledging in the main claims that the receptor-specific response at this concentration remains to be formally characterised by dose-response and antagonist experiments.

7. Alzheimer's framing and translational claim.

The framing of the animal cap as a predictive screening tool for Alzheimer's relevant ligands feels like a stretch from the embryological system being characterised. Xenopus embryonic primary neurons are derived from ectoderm and depend on BMP inhibition and retinoid patterning, whereas adult mammalian neurogenesis involves committed stem-cell niches in a distinct context. While shared pathways may operate, the cross-system bridge is not established here. If feasible (and mammalian dataset available), a cross-species transcriptomic comparison using the existing RNA-seq dataset would substantially strengthen this translational framing. The DEGs identified in Figs 6 and 7 could be compared with published bexarotene-response transcriptomes from mammalian adult neural stem cells and Alzheimer's disease mouse models, using the same overlap framework already applied to the CiliaCarta and AnimalTFDB4 lists. This would provide a quantitative

© 2026. Published by The Company of Biologists under the terms of the Creative Commons Attribution License (https://creativecommons.org/licenses/by/4.0/).

assessment of whether the Xenopus programme recapitulates key features of mammalian bexarotene responses.

Minor/Editing comments:

1. Page 20 line 24, it reads "…cells to be diverted to a neural fate in a process that can be divided into four steps". But those steps are not explicitly numerated afterwards, The Results section also lists "four responses of the noggin/RXRß explants". Can the authors reconcile on these so the reader is able to map on them (if appropriate?)? If they are relevant, what are the 4 steps?

2. Figure and transcriptomic presentation could be simplified. The manuscript contains a large amount of transcriptomic and GO-term analysis, some of which feels repetitive. Simplification of several figures and reduction of lower-priority enrichment analyses (into a supplementary, for example) would improve clarity.

3. Statistical reporting in Fig.3: there are p-values but no n, batch numbers in the figure legend. In B, no mention of test used in the figure legend.

4. Several minor typos/editing errors throughout, for example (but not exclusively):

- Page 2 and page 10 "'BMP-signalling" should read BMP signalling.

- Page 5 line 18 "eg" should read e.g.

- Page 13 line 20 "Trancriptome" should read transcriptome.

- Page 14 line 20 "sox 11" should be sox11

- Page 16 line 21 "nervus" should read nervous.

- Page 11 line 11, missing comma after cyp26a1

- Page 22 line 16-17 "amyloid beta" usually written amyloid-ß

- Various missing spaces before citations. E.g page 10 line 40.

- Make sure it is Pol III (and not Pol II) in page 16 line 18.

- Inconsistencies in spelling : signalling vs signaling; co-injected vs coinjected; 2-fold vs two-fold, Fig X vs Fig. X; down-regulated vs downregulated; RXRß-bexarotene vs RXRß/bexarotene (although this might be on purpose?)

- Citation style inconsistency: "C. Sharpe & Goldstone, 2000a" vs "Sharpe & Goldstone, 2000b"

- Citation style inconsistency for figures . E.g page 8 line 24 and 25 : Fig 3G and Fig. 3G)

- Crossreference of figures in text should be checked. For example, page 17 line 8, Fig. 5A is cited for genes that are discussed in other figures.

- Animal cap culture temperature. Methods mention 21 degrees and figure legend in 3F mentions 22. Please confirm which one (or if both) are correct.

<u>Reviewer 2</u>

Comments for the author

Cardoso et al. test whether bexarotene, a pan-RXR agonist, promotes neurogenesis in Xenopus laevis. They show that RXRß + bexarotene increases the number of Isl1+ embryonic primary neurons (EPNs) in whole embryos and induces neuronal differentiation in Noggin-neuralised animal caps.

© 2026. Published by The Company of Biologists under the terms of the Creative Commons Attribution License (https://creativecommons.org/licenses/by/4.0/).

They then use RNA-seq (3 conditions × 3 replicates) to catalogue differentially expressed genes (DEGs) and propose that bexarotene activates rara expression, "kick-starting" a standard RAR/RXR-atRA-like neurogenic programme.

There are some points that need addressing for clarity and to provide some support for the model the authors have proposed.

1. Stats - Fig. 3A - would an ANOVA with post-hoc tests be more approporiate here?

2. For n's - it would be worth stating how many times teh RT-PCRs were done?

3. Completeness - there are some points in the discussion that are worth revisiting

- The kick-start using RARa is discussed, but it does present some predictions that could be easily tested. If the authors have data on the time course of RARa expression in response to bexatrone, that would be useful to include. Maybe by looking at 2/4/6 hr timepoints, they could ask if RARa precedes neurog2? Without this the authors should acknowledge this limitation.

- The RARa isoforms data should be shown.

- The claim of a dual-function (RXR-only for plaque clearance, RAR/RXR for neurogenesis) is speculative and extends well beyond the Xenopus data presented. The authors might want to temper this statement. Especially given that the plaque clearance data is itself problematic (Cramer et al. (2012) results were hard to reproduce (Fitz et al. 2013, Price et al. 2013, Tesseur et al. 2013, Veeraraghavalu et al. 2013 — multiple groups failed to replicate plaque clearance). The Introduction and Conclusion claim AD benefits without noting this; this may mislead readers into thinking the application is settled when it's contested.

- Clinical trials of bexarotene in AD haven't shown clear benefits, a point that should be mentioned, especially since the discussion uses Xenopus explants as a screening tool for bexarotene-like therapeutics.

- It's a shame that there is no loss-of-function experiment. Would bexarotene still work if RXRß was knocked down? This is an important caveat that should be discussed.

Reviewer's Responses to Questions

Experimental quality
Are experiments performed using appropriate methods that will answer the question (or test the hypothesis or support the observations) posed by the authors? Is the right tool used for the job?
If 'No', please indicate reasons in Comments for Author box below.
Reviewer #1:
- Yes

Reviewer #2:
- Yes

Were the data analyzed using appropriate statistical tests?
If 'No', please indicate reasons in Comments for Author box below.
Reviewer #1:
- Yes

Reviewer #2:
- No

Reproducibility

© 2026. Published by The Company of Biologists under the terms of the Creative Commons Attribution License (https://creativecommons.org/licenses/by/4.0/).

Were experiments performed using adequate number of biological replicates?
If 'No', please indicate reasons in Comments for Author box below.
Reviewer #1:
- Yes

Reviewer #2:
- Yes

Is there sufficient raw data to assess the rigor of the analysis?
If 'No', please indicate reasons in Comments for Author box below.
Reviewer #1:
- No

Reviewer #2:
- Yes

Does the methods section provide sufficient detail to permit reproducibility?
If 'No', please indicate reasons in Comments for Author box below.
Reviewer #1:
- Yes

Reviewer #2:
- Yes

Completeness
Are the manuscript's conclusions supported by the data?
If 'No', please indicate reasons in Comments for Author box below.
Reviewer #1:
- No

Reviewer #2:
- No

Scholarship
Do the authors cite and discuss the merits of data that would argue for and against their conclusion?
If 'No', please indicate reasons in Comments for Author box below.
Reviewer #1:
- Yes

Reviewer #2:
- No

Does the manuscript title & abstract accurately reflect the contents of the manuscript, without hyperbole?
If 'No', please indicate reasons in Comments for Author box below.
Reviewer #1:
- Yes

Reviewer #2:
- Yes

© 2026. Published by The Company of Biologists under the terms of the Creative Commons Attribution License (https://creativecommons.org/licenses/by/4.0/).

First revision

Author response to reviewers' comments

Referees comments

Responses to the Referee's comments are shown in blue.

Page and line numbering refers to the original submission document which now includes the suggested revisions.

Comments from the Reviewers:

Reviewer 1: This manuscript investigates the ability of RXRß/bexarotene signalling to promote neuronal differentiation in BMP-inhibited Xenopus ectoderm and combines embryological manipulation with transcriptomic analysis to define the underlying transcriptional programme. The study identifies temporal requirements for RXR signalling, demonstrates induction of early primary neurons in animal cap explants, and characterises associated changes in gene expression linked to neural processes.

The animal cap explant assay is presented as a tractable screening platform for retinoid-related neurogenic ligands, and the transcriptomic dataset will be of value to the Xenopus and retinoid-signalling communities. The RNA-seq component substantially strengthens the manuscript by moving beyond a descriptive phenotype toward mechanistic interpretation. The manuscript contains several valuable observations that will likely be of interest to researchers studying neural induction, retinoid signalling, and Xenopus developmental systems. The limitations section is honest: it acknowledges the missing atRA RNA-seq dataset and the absence of functional validation.

However, the mechanistic conclusions frequently go beyond what the transcriptomic data alone can support, including the central claim that bexarotene and atRA treatments act through related pathways. The manuscript also has issues with clarity and paragraph density, and contains a noticeable number of typos and formatting errors.

Major general comments:

Throughout the text, mechanistic claims should either be experimentally strengthened with **validation (proposed below) or softened in the writing**. As it stands, the discussion's mechanistic claims are stronger than the data here support. Reframing the manuscript more explicitly as a developmental transcriptomic resource paper, rather than a definitive signalling mechanism paper, would resolve much of this without requiring new experiments.

The manuscript has been 'softened in the writing' and now emphasises the specific transcriptomic findings. Comments on signalling mechanisms have been reduced (see below).

The manuscript would also benefit substantially from paragraph restructuring and more careful punctuation. Comprehension is often hindered by dense paragraphs (>10 lines) that contain multiple concepts, interpretations, and gene catalogues. This causes reader fatigue and makes it difficult to identify what is central. One example is the section on page 13-14 starting "Considering these 294…", it would aid the reader if these type of sections are split into shorter paragraphs.

The Results relating to the transcriptomic analysis was originally two sections. This has now been restructured and the transcriptomic results split into four shorter sections with shorter paragraphs:

- Transcriptome analysis of isolated ectodermal explants identifies four responses to the expression of Noggin and RXRb mRNA in explants.
- Noggin-neuralised animal caps with enhanced RXR□-bexarotene signalling downregulate genes associated with anterior neural development.
- Genes whose enhanced expression is maintained in response to Noggin/RXR□ mRNA with or without bexarotene include those that balance the processes of proliferation and differentiation.

© 2026. Published by The Company of Biologists under the terms of the Creative Commons Attribution License (https://creativecommons.org/licenses/by/4.0/).

- Genes upregulated when Noggin/RXRb mRNA injected animal caps are treated with bexarotene promote neuronal differentiation and contribute to anterior-posterior identity.

Major specific comments:

1. The "kick-start" model is speculative and not supported by the current data.

The proposed model in which RXRß/bexarotene signalling induces rara and thereby kick-starts canonical retinoid signalling is interesting but remains untested. The authors note increased rara expression but do not separate cause from correlation, and there is no direct evidence presented that RAR activity is required for the bexarotene response (if such evidence is in the manuscript, it should be highlighted more clearly).

The RNA-seq experiment never compares Noggin/RXRß + bexarotene with Noggin/RAR/RXR + atRA. Neuronal differentiation in Noggin + RXRß + bexarotene caps (Fig. 4) could in principle be driven by RXR partnerships other than RAR. The induction of Wnt and FGF pathway components (Fig. 7) could likewise indicate a parallel or alternative route to neurogenesi

I would ask the authors either to **soften this model in both text and figure**, or to provide functional validation. For softening: Fig. 9 should be explicitly labelled "Proposed model/hypothesis" in the title, with an open question in the rara arrow (e.g. question mark/dashed arrow). For validation, possible options include inhibition of RAR signalling during RXRß/bexarotene treatment or an RNA-seq comparison of Noggin/RXRß + bexarotene with Noggin/RAR/RXR + atRA. I appreciate validating is a substantial ask. Low-cost bulk RNA-seq (e.g Plasmidsaurus) would make this more tractable if the authors wish to pursue it, but reframing the claims is an acceptable alternative.

This model has been softened both in the text (p26) and figure 9 and no longer refers to a 'kick-start' mechanism. The title of Fig. 9 now states that this is a proposed model and, as suggested, the steps leading to the activation of retinoid signalling and then the activation of retinoid target genes leading to the formation of neuronal cells are shown as dashed arrows in grey (rather than solid black arrows for the validated steps). In the text we now refer to Fig. 9 as a 'hypothetical model' (p26 l26).

2. The rara2 isoform claim should not be relegated to "data not shown".

The specific upregulation of the rara2 isoform could be a piece of evidence for the kick-start model. A simple read-coverage plot from the RNA-seq across the rara locus, would substantially strengthen this claim without requiring new experiments.

A read coverage plot (Sashimi plot) for the rara2 locus has been included as part B in Fig. 7.

3. HNK1 as a neuronal marker (Fig. 4).

HNK1 is also expressed by neural crest derivatives in Xenopus, so HNK1 positivity alone does not unambiguously identify neurons. **I suggest the authors acknowledge that their assay shows HNK1-positive cells consistent with neuronal differentiation but requiring additional markers (or co-staining) to definitively confirm neuronal identity**. It would also be helpful to briefly mention why animal caps were paired in this experiment. Additionally, the blue background of panels B-G makes the ßgal signal difficult to see in print; could the authors adjust contrast or LUTs to improve ßgal visibility?

The specificity of the anti-HNK1 monoclonal has been changed to state that it recognises both embryonic primary neurons and some neural crest cells both in the Methods section (p30 l37) and in the Results section (p9 l33/34) along with the comment that recognition of HNK-1 staining is 'consistent' with neuronal identity.

In the Methods section we now comment that animal caps are paired as this practice enhances survival of the explants as the outer ectodermal coat is thought to reform more easily along the

© 2026. Published by The Company of Biologists under the terms of the Creative Commons Attribution License (https://creativecommons.org/licenses/by/4.0/).

join between two opposed caps than when a single cap rolls into a ball. It is not uncommon to do this when dealing with animal cap explants (p29 l32).

The contrast in figure 4 sections B-G has been adjusted solely to enhance the distinction between the background and the bgal signal.

4. Cell-type resolution in the bexarotene response (Fig. 7).
Bulk RNA-seq cannot determine whether the posterior patterning and Wnt-related transcriptional changes occur within neural precursor cells specifically, or in non-neural subpopulations of the explants. It also cannot resolve whether cdx/hox and Wnt induction co-occur in the same cells. Without single-cell or spatial validation by ISH, the attribution of posterior character specifically to the neurogenic lineage remains an inference, and this should be acknowledged in the text.
In the Discussion we now comment: 'Since these observations relate to the explants, it would be interesting to determine how the expression of posterior patterning and Wnt signalling genes relates to individual cells within the subpopulations of neural precursor and non-neural cells.' (p26 l8).

5. Sensitivity of the canonical Wnt enrichment to the DEG cut-off.
The DEG analysis uses a stringent cut-off and canonical Wnt is then highlighted via GO/pathway over-representation. This inference may be sensitive to the chosen threshold and could miss more modest but coordinated changes. I suggest the authors repeat the over-representation analysis across multiple DEG cut-offs and summarise whether canonical Wnt enrichment persists (confirming it is not an artefact of stringency) across the three replicates. It would also be interesting to know whether non-canonical Wnt components show coordinated changes across the three replicates at lower threshold.
The pathway analysis has now been performed at three different stringencies. At each of the stringencies (both higher and lower than the original), Wnt, cadherin and Alzheimers disease remain the primary pathways identified, and these have an extensive overlap of genes. There was no indication of non-canonical Wnt signalling in any of the analyses.

Figure provided in confidence for the referees has been removed.

6. Bexarotene concentration and specificity.

All bexarotene experiments use a single 100 nM concentration, without dose-response characterisation, receptor-selective antagonist controls, or assessment of off-target actions. The authors note in both introduction and discussion that bexarotene specificity has been questioned. I thus wonder why this is the dose chosen by the authors. This could be addressed through text edits. Perhaps noting that the chosen concentration is consistent with the mammalian bexarotene literature already cited (if so), and acknowledging in the main claims that the receptor-specific response at this concentration remains to be formally characterised by dose-response and antagonist experiments.

This is now addressed in the Methods and papers that set a precedent are referenced. Bexarotene is generally used between 30nM and 1mM. We also comment that 100nM bexarotene does not affect the survival rate of *Xenopus laevis* embryos to the tailbud stage. (p29 l13).

7. Alzheimer's framing and translational claim.

The framing of the animal cap as a predictive screening tool for Alzheimer's relevant ligands feels like a stretch from the embryological system being characterised. Xenopus embryonic primary neurons are derived from ectoderm and depend on BMP inhibition and retinoid patterning, whereas adult mammalian neurogenesis involves committed stem-cell niches in a distinct context. While shared pathways may operate, the cross-system bridge is not established here. If feasible (and mammalian dataset available), a cross-species transcriptomic comparison using the existing RNA-seq dataset would substantially strengthen this translational framing. The DEGs identified in Figs 6 and 7 could be compared with published bexarotene-response transcriptomes from mammalian adult neural stem cells and Alzheimer's disease mouse models, using the same overlap framework already applied to the CiliaCarta and AnimalTFDB4 lists. This would provide a quantitative

© 2026. Published by The Company of Biologists under the terms of the Creative Commons Attribution License (https://creativecommons.org/licenses/by/4.0/).

assessment of whether the Xenopus programme recapitulates key features of mammalian bexarotene responses.

This is not the impression we wished to give, we do not see the animal cap system as a predictive screening tool for Alzheimer's relevant ligands. Rather, we see the system as an efficient means of testing the ability of synthetic retinoid agonists to promote neurogenesis. For example , beyond the scope of this paper, it would be interesting to test whether non-retinoid agonists of RXR such as gamma linolenic acid can also promote neurogenesis.

To remove any ambiguity, from the Introduction we have removed the sentence 'The simple animal cap assay provides a rapid and effective method to test the potential of nuclear receptor agonists to promote neurogenesis.' (p4 l2). In the Discussion we emphasise '…the ability of bexarotene to promote neuron formation using *Xenopus laevis* ectodermal explants as a rapid and accessible tool to examine the role of nuclear receptors in neurogenesis' as a key point of the paper (p28 l42).

We agree that there are significant differences between mammalian neurogenesis and the formation of embryonic primary neurons and comment on this in the Discussion. We are not aiming in this paper to directly compare neurogenesis in these two systems and whilst a comparison between the DEGs described here and those identified in a mammalian neural system after bexarotene treatment would be fascinating, it is beyond the scope of this analysis.

Minor/Editing comments:

1. Page 20 line 24, it reads "…cells to be diverted to a neural fate in a process that can be divided into four steps". But those steps are not explicitly numerated afterwards, The Results section also lists "four responses of the noggin/RXRß explants". Can the authors reconcile on these so the reader is able to map on them (if appropriate?)? If they are relevant, what are the 4 steps?

The four steps are detailed and numbered in the last paragraph of the Results section 'Transcriptome analysis of isolated ectodermal explants identifies four responses to the expression of Noggin and RXRb mRNA in explants.' (p13 l3). In the Discussion the four steps are now recapped: 'The formation of EPNs first requires ectodermal cells to be diverted to a neural fate in a process that can be divided into four steps: the inhibition of BMP signalling; the consequent suppression of mucociliary epidermal development; the downregulation of inhibitors of neural development such as ventx2 and the promotion of a proliferative neural precursor state.' (p25 l24)

2. Figure and transcriptomic presentation could be simplified. The manuscript contains a large amount of transcriptomic and GO-term analysis, some of which feels repetitive. Simplification of several figures and reduction of lower-priority enrichment analyses (into a supplementary, for example) would improve clarity.

We agree, it is always difficult to know which data to include. We have revised three figures by removing some data on GO-term analysis. This data is now presented as three Supplementary figures. In the submitted 'original manuscript with highlighted changes' we have included both the original and revised figures to illustrate the panels removed.

Fig. 5 GO term graph Fig. 5H has been removed and is now Fig. S1

Fig. 6 the GO term analyses: Fig. 6B and Fig. 6F have been removed and are now Fig. S2 and Fig. S3

Fig. 7 The Go term analysis: Fig 7B has now been removed and is now Fig. S4

3. Statistical reporting in Fig.3: there are p-values but no n, batch numbers in the figure legend. In B, no mention of test used in the figure legend.
We have now included n values either in the figure legends or in the Methods section for all relevant figures. Where appropriate, the statistics have been recalculated (as suggested by Referee 2) using ANOVA and post-hoc analysis.

4. Several minor typos/editing errors throughout, for example (but not exclusively):
- Page 2 and page 10 "'BMP-signalling" should read BMP signalling.

© 2026. Published by The Company of Biologists under the terms of the Creative Commons Attribution License (https://creativecommons.org/licenses/by/4.0/).

Done

- Page 5 line 18 "eg" should read e.g.

Done

- Page 13 line 20 "Trancriptome" should read transcriptome.

Done

- Page 14 line 20 "sox 11" should be sox11

Done

- Page 16 line 21 "nervus" should read nervous.

Done

- Page 11 line 11, missing comma after cyp26a1

Done

- Page 22 line 16-17 "amyloid beta" usually written amyloid-ß

Done

- Various missing spaces before citations. E.g page 10 line 40.

Done

- Make sure it is Pol III (and not Pol II) in page 16 line 18.

Done – it is PolII (pol2)

- Inconsistencies in spelling : **signalling** vs signaling; **co-injected** vs coinjected; 2-fold vs **two-fold** , Fig X vs Fig. X ; down-regulated vs **downregulated**;

We have used a document-wide 'find and replace' to remove these inconsistencies.

RXRß-bexarotene vs RXRß/bexarotene (although this might be on purpose?)

This is now consistently RXRß-bexarotene.

- Citation style inconsistency: "C. Sharpe & Goldstone, 2000a" vs "Sharpe & Goldstone, 2000b"

Citations and reference list constructed using Mendeley. Have removed the intials. (On some papers I am C.Sharpe and in others C.R. Sharpe and this is the cause of the error in Mendeley).

- Citation style inconsistency for figures . E.g page 8 line 24 and 25 : Fig 3G and Fig. 3G)

Consistent style applied

- Crossreference of figures in text should be checked. For example, page 17 line 8, Fig. 5A is cited for genes that are discussed in other figures.

Checked and corrected

- Animal cap culture temperature. Methods mention 21 degrees and figure legend in 3F mentions 22. Please confirm which one (or if both) are correct.

© 2026. Published by The Company of Biologists under the terms of the Creative Commons Attribution License (https://creativecommons.org/licenses/by/4.0/).

21 is correct – this has been changed in the figure legend.

Reviewer 2: Cardoso et al. test whether bexarotene, a pan-RXR agonist, promotes neurogenesis in Xenopus laevis. They show that RXRß + bexarotene increases the number of Isl1+ embryonic primary neurons (EPNs) in whole embryos and induces neuronal differentiation in Noggin-neuralised animal caps. They then use RNA-seq (3 conditions × 3 replicates) to catalogue differentially expressed genes (DEGs) and propose that bexarotene activates rara expression, "kick-starting" a standard RAR/RXR-atRA-like neurogenic programme.

There are some points that need addressing for clarity and to provide some support for the model the authors have proposed.

1. Stats - Fig. 3A - would an ANOVA with post-hoc tests be more approporiate here?

In Fig. 3A we are making 1:1 comparisons (left side untreated v left side treated and then right side untreated against right side treated). We feel that a basic Student's t test is appropriate in this case, though would be happy to do an ANOVA with post-hoc tests if this is incorrect.

In the other panels (Fig. 3B and Fig. 3G) we are making multiple comparisons and in these cases we agree that an ANOVA is more appropriate. An ANOVA with Tukey's HSD test has now been applied to the data in these panels. The significance levels change a little, but do not affect the conclusions.

2. For n's - it would be worth stating how many times the RT-PCRs were done?

This is now stated in the Method section relating to RT-PCR. (p30 l8)

3. Completeness - there are some points in the discussion that are worth revisiting

- The kick-start using RARa is discussed, but it does present some predictions that could be easily tested. If the authors have data on the time course of RARa expression in response to bexatrone, that would be useful to include. Maybe by looking at 2/4/6 hr timepoints, they could ask if RARa precedes neurog2? Without this the authors should acknowledge this limitation.

This is now acknowledged in the Limitations section (p28 l16).

- The RARa isoforms data should be shown.

A read coverage plot (Sashimi plot) for the rara2 locus has been included as part B in Fig. 7.

- The claim of a dual-function (RXR-only for plaque clearance, RAR/RXR for neurogenesis) is speculative and extends well beyond the Xenopus data presented. The authors might want to temper this statement. Especially given that the plaque clearance data is itself problematic (Cramer et al. (2012) results were hard to reproduce (Fitz et al. 2013, Price et al. 2013, Tesseur et al. 2013, Veeraraghavalu et al. 2013 — multiple groups failed to replicate plaque clearance). The Introduction and Conclusion claim AD benefits without noting this; this may mislead readers into thinking the application is settled when it's contested.

We now make it clear in the Introduction (p3 l12) and Conclusion (p28 l23) that the idea that bexarotene reduces plaque is still speculative. However, we feel that a paper concerning bexarotene and RXR needs to mention this possibility and we now do so in a more cautious manner.

- Clinical trials of bexarotene in AD haven't shown clear benefits, a point that should be mentioned, especially since the discussion uses Xenopus explants as a screening tool for bexarotene-like therapeutics.

We now include a reference to the clinical trials in Conclusion (p28 l28) as part of a balanced commentary on the role of bexarotene.

© 2026. Published by The Company of Biologists under the terms of the Creative Commons Attribution License (https://creativecommons.org/licenses/by/4.0/).

- It's a shame that there is no loss-of-function experiment. Would bexarotene still work if RXRß was knocked down? This is an important caveat that should be discussed.

This paper uses a gain of function analysis to look at RXRb-bexarotene activity. An alternative would indeed be to knock down the expression of RXRb and ask whether bexarotene can still promote the formation of additional neurons. This approach would be likely, however, to cause additional effects beyond neurogenesis as heterodimerisation with an RXR protein is a requisite for the activity of many of the type II nuclear receptors. It is also possible that one of the other RXR proteins (a or g) might compensate for the loss of RXRb. This is now commented on in the Discussion (p26 l22).

Reviewer's Responses to Questions

Experimental quality

Are experiments performed using appropriate methods that will answer the question (or test the hypothesis or support the observations) posed by the authors? Is the right tool used for the job?
If 'No', please indicate reasons in Comments for Author box below.
Reviewer #1:

- Yes

Reviewer #2:

- Yes

Were the data analyzed using appropriate statistical tests?
If 'No', please indicate reasons in Comments for Author box below.
Reviewer #1:

- Yes

Reviewer #2:

- No

Reproducibility
Were experiments performed using adequate number of biological replicates?
If 'No', please indicate reasons in Comments for Author box below.
Reviewer #1:

- Yes

Reviewer #2:

- Yes

Is there sufficient raw data to assess the rigor of the analysis?
If 'No', please indicate reasons in Comments for Author box below.
Reviewer #1:

- No

Reviewer #2:

- Yes

Does the methods section provide sufficient detail to permit reproducibility?
If 'No', please indicate reasons in Comments for Author box below.
Reviewer #1:

- Yes

© 2026. Published by The Company of Biologists under the terms of the Creative Commons Attribution License (https://creativecommons.org/licenses/by/4.0/).

Reviewer #2:
- Yes

Completeness
Are the manuscript's conclusions supported by the data?
If 'No', please indicate reasons in Comments for Author box below.
Reviewer #1:
- No

Reviewer #2:
- No

Scholarship
Do the authors cite and discuss the merits of data that would argue for and against their conclusion?
If 'No', please indicate reasons in Comments for Author box below.
Reviewer #1:
- Yes

Reviewer #2:
- No

Does the manuscript title & abstract accurately reflect the contents of the manuscript, without hyperbole?
If 'No', please indicate reasons in Comments for Author box below.
Reviewer #1:
- Yes

Reviewer #2:
- Yes

Second decision letter

MS ID#: bio.062675R1

MS Title: **Signaling through RXRß and its agonist, bexarotene, promotes neuron formation in Xenopus laevis embryos.**

Authors: Daniela Lopes Cardoso; Marilena Loizou; Danai D. Fourla; Charles Woodman; Matthew J. Guille; Colin R. Sharpe

I am happy to tell you that your manuscript has been accepted for publication in Biology Open, pending our standard publication integrity checks. It was accepted on 15th June 2026.

© 2026. Published by The Company of Biologists under the terms of the Creative Commons Attribution License (https://creativecommons.org/licenses/by/4.0/).
